# HVEM-LIGHT signaling promotes antibody-dependent neutrophil Fc**γ**R-mediated trogocytosis against herpes simplex virus infection

**DOI:** 10.1172/JCI203771

**Published:** 2026-06-04

**Authors:** Matthew S. Gromisch, Masayuki Kuraoka, Carl F. Ware, Steven C. Almo, Betsy C. Herold

**Affiliations:** 1 Department of Microbiology and Immunology, Albert Einstein College of Medicine, New York, New York, USA.; 2 Department of Immunology, Duke University School of Medicine, Durham, North Carolina, USA.; 3 Infectious and Inflammatory Diseases Research Center, Sanford Burnham Prebys Medical Discovery Institute, La Jolla, California, USA.; 4 Department of Biochemistry and; 5 Department of Pediatrics, Albert Einstein College of Medicine, New York, New York, USA.

**Keywords:** Immunology, Infectious disease, Virology, Adaptive immunity, Neutrophils, Vaccines

## Abstract

Studies with a candidate vaccine deleted in glycoprotein D (ΔgD-2) for herpes simplex virus (HSV) prevention uncovered a role for herpes virus entry mediator (HVEM) in mediating antibody-dependent cell-mediated killing (ADCK) of virally infected cells. Antibodies elicited by ΔgD-2 passively protect WT but not Fc γ receptor (FcγR) or HVEM knockout (KO) mice. The goals of this study were to identify which cells mediate ADCK and the role of HVEM signaling. Using HVEM ligand and conditional cell-type–specific HVEM-KO mice combined with in vitro mouse and human cytolytic assays, we demonstrate that ADCK of HSV-infected cells is mediated primarily by neutrophils and requires their expression of HVEM and its ligand, LIGHT. Cytolysis is not associated with granzyme and perforin production but occurs by a trogocytosis-like pathway. Pharmacological inhibition of myosin light-chain kinase (MLCK), which mediates trogocytosis, inhibits cytolysis. Similar results were obtained when human neutrophils were cocultured with HSV-infected cells opsonized with ADCK-containing human immune serum or with breast cancer cells treated with an anti-HER2 trogocytosis mediating antibody. Killing was significantly reduced when an MLCK inhibitor or blocking antibodies to CD16a, HVEM, or LIGHT were added. Together, these results define a mechanism of HVEM-enhanced FcγR-mediated neutrophil-dependent ADCK of targets cells.

## Abstract 2

This study identifies a critical role for neutrophils in mediating antitbody-dependent killing of HSV infected cells by a trogocytic mechanism.

## Introduction

Herpes simplex viruses type 1 and 2 (HSV-1 and HSV-2) pose major global health burdens, and, to date, no effective prophylactic or therapeutic vaccines exist (1, 2). Vaccine efforts have primarily focused on generating neutralizing antibodies (nAbs) targeting viral glycoprotein D (gD). However, these approaches have failed to provide substantial efficacy in phase 3 clinical trials (3–6). Retrospective analyses of vaccine immunogenicity suggests that the failures may reflect the ability of virus to escape nAbs and the need to also elicit cytolytic responses including antibodies that mediate antibody-dependent cell-mediated killing (ADCK) (7, 8). Interactions between Fc-γ receptors (FcγRs) expressed by NK cells, macrophages/monocytes, dendritic cells, and neutrophils and the Fc component of antibodies bound to their antigenic targets on infected cells can mediate killing by multiple mechanisms (9–15). These include antibody-dependent cellular cytolysis (ADCC), which classically involves the release of cytotoxic granules including granzymes and perforin, and antibody-dependent phagocytosis (11, 16). Other mechanisms include release of cytotoxic cytokines such as tumor necrosis factor (TNF) and Fas ligand and production of reactive oxygen species (ROS) or their intermediates (17–19). More recent studies have shown that FcγRs on neutrophils can also kill through trogocytosis, a process whereby the neutrophil extracts fragments of the antibody-coated target cell membrane resulting in cell death (14, 20–22).

The importance of cytolytic antibodies was highlighted by mouse model studies with a single-cycle HSV-2 viral vaccine candidate deleted in gD, designated ΔgD-2. ΔgD-2 is grown on an HSV-1 gD-expressing cell line (VD60) to yield viral particles phenotypically complemented with gD-1 on their envelope but genetically gD null and is therefore restricted to a single cycle of infection because gD is required for entry and cell-to-cell spread. Immunization of mice with ΔgD-2 resulted in complete protection against clinical isolates of HSV-1 and HSV-2 following vaginal, skin, ocular, or neonatal challenge in female and/or male mice, a response that was significantly greater compared with results obtained with adjuvanted gD protein or other vaccines (23–29). However, in contrast with the neutralizing antibody response characteristic of gD containing vaccines or acute HSV infection, ΔgD-2 elicited a predominant ADCC response. Passive transfer of immune serum from ΔgD-2-vaccinated but not gD protein-vaccinated mice completely protected WT mice from lethal viral challenge. However, protection was lost when immune serum was transferred into FcγRIV-knockout mice, the receptor primarily responsible for mediating ADCC (23–27).

Several clinical studies support a role for ADCC in HSV protection. First, both an MF59-adjuvanted gD and glycoprotein B (gB) protein vaccine and a differently adjuvanted (AS04) gD-only protein vaccine elicited neutralizing but no ADCC responses; both vaccines failed to prevent HSV-2 in clinical trials (3, 4). Second, the immune response to primary HSV infection is dominated by nAbs with little ADCC detected within the first year, the time frame when viral recurrences are most common (8). Similar results were observed in a mouse model of recurrent disease (30). Third, placentally transferred ADCC-mediating HSV-specific Abs were associated with protection against disseminated neonatal HSV disease after controlling for nAb titers in clinical studies (31).

The ability of virus to escape nAbs but not cytolytic antibodies coupled with the observation that ΔgD-2 but not primary HSV-infection or gD subunit protein vaccines elicit ADCC responses, suggested that gD might interfere with ADCC via its interactions with herpes virus entry mediator (HVEM), a member of the TNF receptor superfamily (TNFRSF14). HVEM is an immunomodulatory molecule expressed by multiple cell types, including lymphoid and myeloid cells (32–40). HVEM binds to one of several ligands to elicit costimulatory or inhibitory responses, and gD blocks these interactions (34, 41–43). Interactions with LIGHT, a TNF superfamily ligand, provide a costimulatory signal (44); interactions with BTLA, an immunoglobulin superfamily protein, provide an inhibitory signal (45), and binding to CD160, a GPI-anchored member of the immunoglobulin superfamily, typically provides costimulatory signaling to NK cells but can also elicit inhibitory responses in CD8^+^ T cells via engagement of MHC-I (46, 47). These ligands also have the capability to interact with HVEM either in cis (same cell) or in trans (different cell). Ligands binding to HVEM in trans are typically costimulatory whereas cis interactions with BTLA and/or CD160 usually mediate coinhibitory signaling (48). The existence of HVEM/LIGHT cis interactions has yet to be documented. Glycoprotein D binds to HVEM and blocks its interactions with its natural ligands but a role for HVEM and its ligands in mediating ADCC had not been previously identified.

To test whether HVEM signaling contributes to ADCC, we passively transferred immune serum from ΔgD-2–vaccinated WT mice (replete with ADCC-mediating Abs), into *Hvem*^–/–^ mice. The immune serum failed to protect the knockout (KO) mice. Moreover, addition of soluble gD protein (5–10μg) or HVEM blocking antibodies significantly reduced FcγR NFAT activation in an ADCC reporter bioassay with either mouse or human anti-HSV immune serum treated virally infected cells. Conversely, ADCC activity was increased if the targets were infected with ΔgD-2 compared with WT virus. HVEM blocking antibodies also reduced the response in the ADCC reporter assay when the targets were anti-CD20 (rituximab)–treated Raji cells, indicating a more generalizable role for HVEM signaling in mediating ADCC (27). Building on these observations, the goals of this study were to identify the immune cells, HVEM ligands, and underlying mechanism involved in promoting antibody-dependent killing of HSV-infected cells.

## Results

### LIGHT is the primary HVEM ligand involved in promoting antibody-dependent killing.

**Figure 1:**
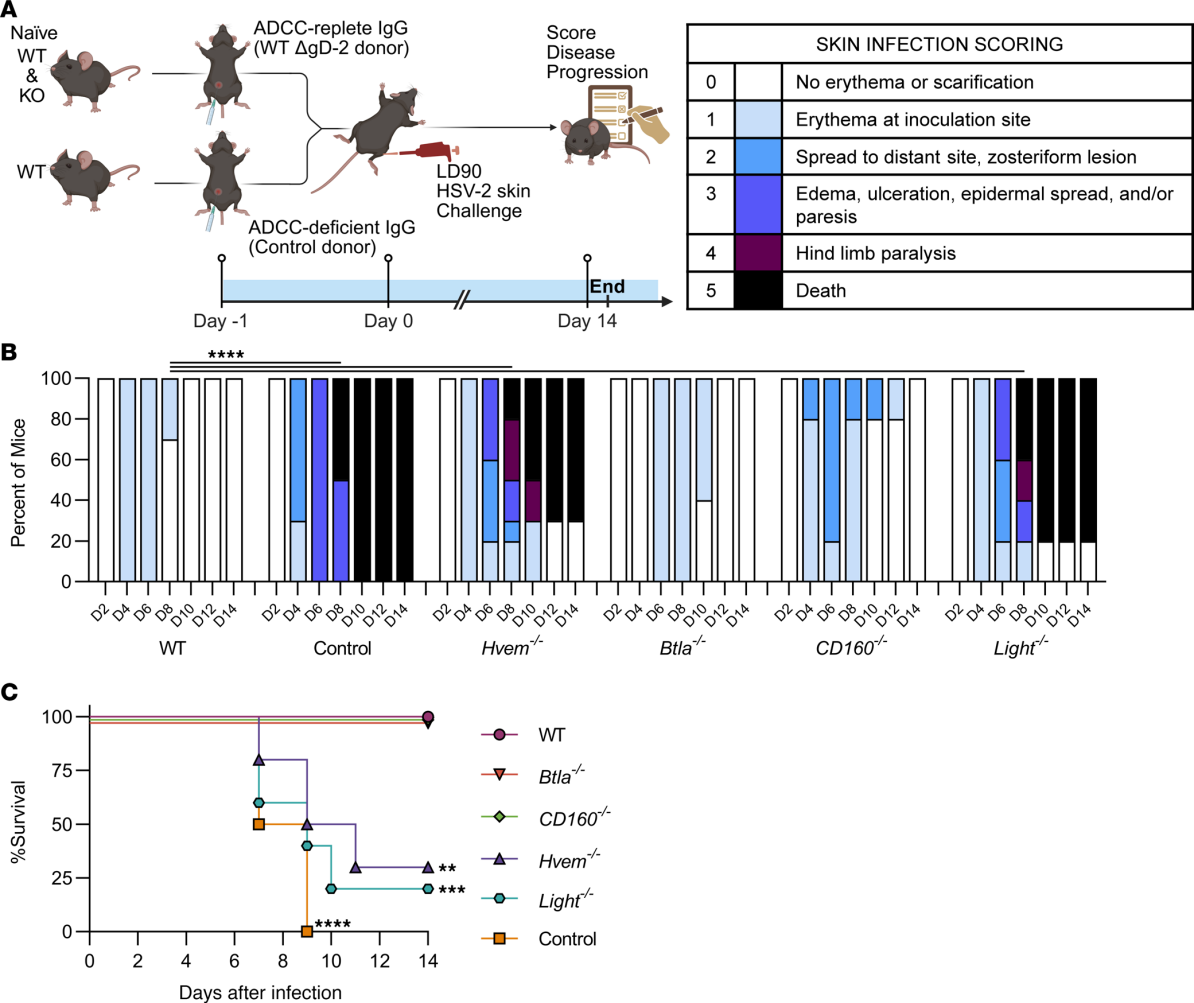
HVEM-LIGHT signaling promotes protective ADCC mediation against HSV. (**A**) WT and the indicated knockout strains of mice were intraperitoneally administered serum containing 750 μg of IgG isolated from ΔgD-2 vaccinated mice one day prior to challenge on the skin with a lethal dose of HSV-2 (4674). As an additional control, WT mice were administered serum containing 750 μg of IgG isolated from control-vaccinated mice immunized with a VD60 cell lysate. Panel created using BioRender. (**B**) Mice were scored daily for signs of disease (23, 24, 28) and (**C**) percentage survival following challenge; scores were compared by 2-way ANOVA with Šidák’s multiple comparison test (****P < 0.0001). Survival was compared with WT recipient mice by Gehan-Breslow-Wilcoxon test (**P < 0.01, ***P < 0.001, ****P < 0.0001). *n* = 10 mice per group from 2 independent experiments.

To identify which of the HVEM ligands contribute to the passive immune protection observed with ΔgD-2 immune serum, we intraperitoneally administered pooled immune serum containing 750 μg of total IgG isolated from ΔgD-2- or control vaccinated mice into naive C57BL/6 (B6) WT, *Hvem^–/–^*, *Btla*^–/–^, *CD160*^–/–^, or *Light*^–/–^ mice 24 hours before challenging the mice on the skin with a lethal dose of HSV-2(4674), a clinical isolate (Figure 1A). We quantified the total IgG and the HSV-specific IgG by ELISA as well as the fold-activation of mouse FcγRIV (ADCC biomarker assay) in the pooled immune serum prior to transfer studies (Supplemental Figure 1, A–C; supplemental material available online with this article; https://doi.org/10.1172/JCI203771DS1). Mice were monitored and scored daily for signs of disease. The WT, *Btla^–/–^* and *CD160^–/–^* mice survived with minimal signs of disease and full recovery between days 10–14, whereas both the *Hvem^–/–^* and *Light^–/–^* mice showed significant loss of protection (*P* < 0.0001) with only 20%–30% survival (Figure 1, B and C).

### Identification of cell subpopulations expressing HVEM, LIGHT, and FcγRIV as potential mediators of antibody-dependent cytolytic activity.

**Figure 2:**
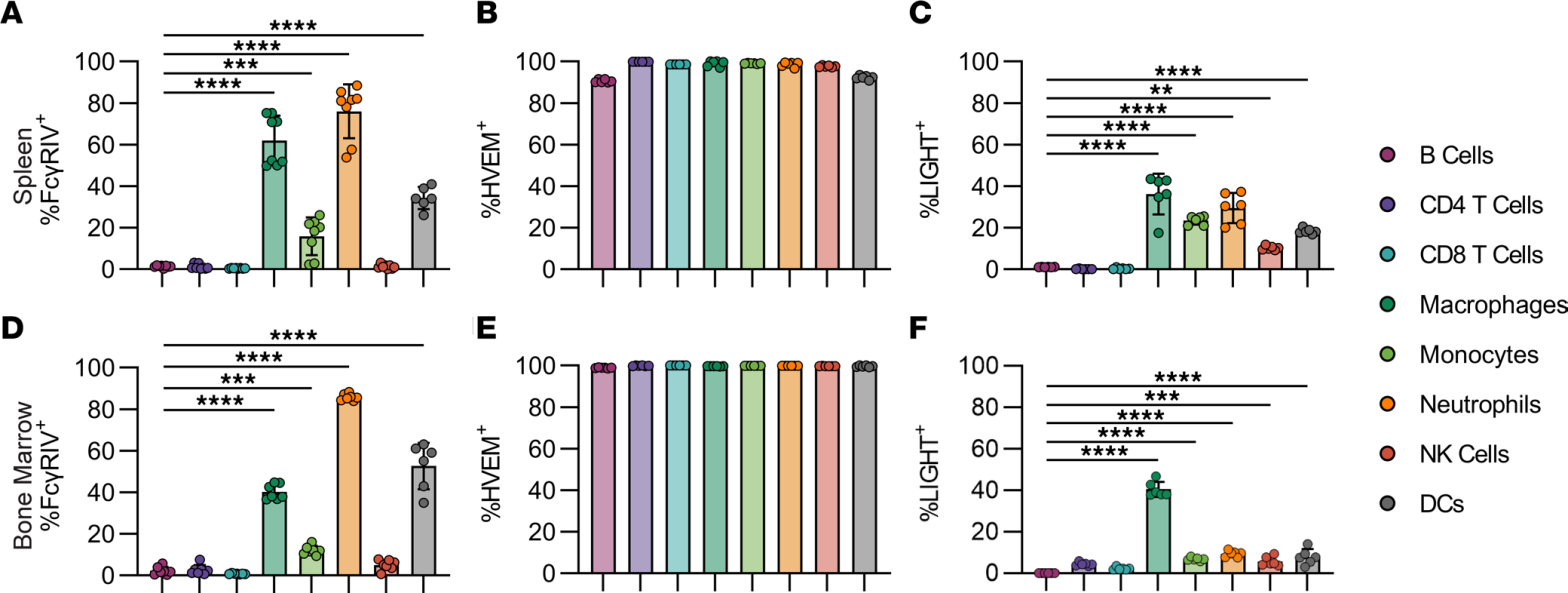
Neutrophils, monocytes/macrophages, and DCs express FcγRIV, HVEM, and LIGHT. Cells isolated from WT mouse splenocytes (**A**–**C**) or bone marrow (**D**–**F**) were stained with immune cell markers and for expression of FcγRIV (**A** and **D**), HVEM (**B** and **E**), and LIGHT (**C** and **F**) and analyzed by flow cytometry. FcγRIV and LIGHT expression for neutrophils (CD11b^+^Ly6G^+^), macrophages (CD11b^+^F4/80^+^), a smaller percentage of monocytes (CD11b^+^Ly6C^+^Ly6G^–^F4/80^–^), and dendritic cells (CD11c^+^MHCII^+^F4/80^–^) were compared with B cells (CD19^+^), which express neither FcγRIV or LIGHT (40, 49) by 1-way ANOVA with Holm-Šidák’s multiple comparisons (**P < 0.01, ***P < 0.001, and ****P < 0.0001; *n* = 6).

To identify which cells mediated the antibody-dependent killing, we generated single-cell suspensions of immune cells from the spleen or bone marrow of WT mice and assessed the expression of FcγRIV, HVEM, and LIGHT in different cell subpopulations by flow cytometry (Supplemental Figure 2). Most of the neutrophils (CD11b^+^Ly6G^+^), macrophages (CD11b^+^F4/80^+^), a smaller percentage of monocytes (CD11b^+^Ly6C^+^Ly6G^–^F4/80^–^), and dendritic cells (CD11c^+^MHCII^+^F4/80^–^) isolated from spleen or bone marrow but not B cells, CD4^+^ T cells, or CD8^+^ T cells expressed FcγRIV. The mouse NK cells also expressed little FcγRIV, a finding consistent with other studies (49) (Figure 2, A and D). All cell subpopulations isolated from both spleen and bone marrow uniformly express high levels of HVEM (Figure 2, B and E). LIGHT expression, however, was restricted in the spleen to neutrophils, macrophages, monocytes, dendritic cells, and was weakly expressed by NK cells (Figure 2C). These findings were consistent with scRNA data publicly available from the Immunological Genome Project (ImmGen) (50). Similar results for FcγRIV and HVEM were obtained with cells isolated from the bone marrow, and, except for macrophages, LIGHT expression was lower in bone marrow compared with spleen (Figure 2F). These data demonstrate that neutrophils, macrophages, monocytes, and dendritic cells are candidates for mediating antibody-dependent killing against HSV.

### Immune cells generate reactive oxygen and nitrogen species (RNOS) but not granzyme or perforin in antibody-dependent killing assays.

**Figure 3:**
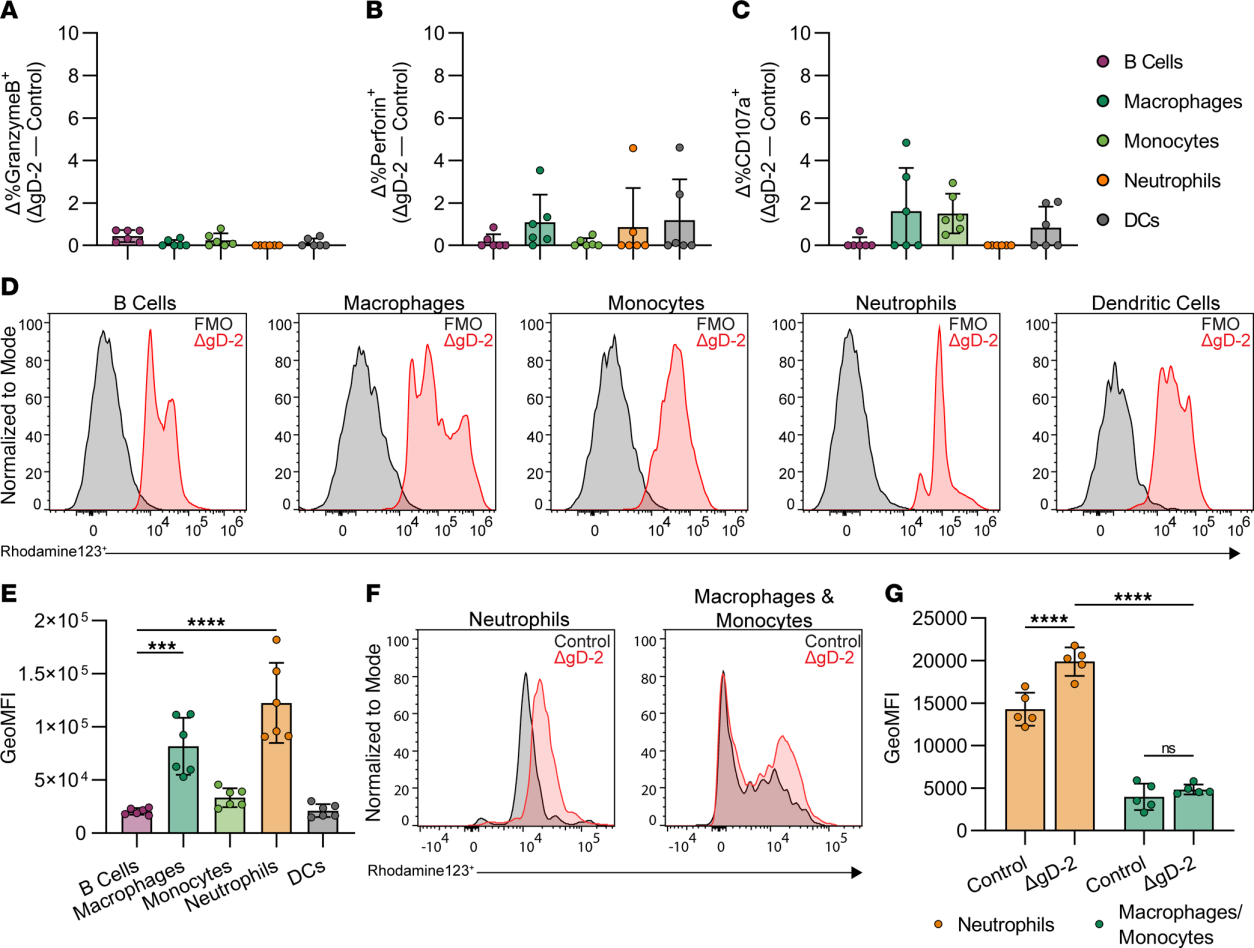
Neutrophils generate RNOS but not granzyme or perforin when cocultured with antibody-coated HSV-infected target cells. Immune cells isolated from bone marrow of WT mice were incubated for 4 hours with HSV-2–infected Vero cells (targets) that had been pretreated with control or ΔgD-2 immune serum. The percentage of immune cells staining positive for intracellular granzyme B (**A**) or perforin (**B**) or cell surface CD107a (**C**) after coculture with ΔgD-2 serum–opsonized targets minus control-opsonized targets (*n* = 6). (**D**) Representative flow plots of rhodamine 123 expression of indicated immune cell populations either with (red/orange) or without (black) DHR123 staining following incubation with ΔgD-2 serum–opsonized targets (*n* = 6). (**E**) Geometric mean fluorescent intensity (GeoMFI) of Rhodamine123 staining in represented immune cell populations isolated from total bone marrow cocultured with ΔgD-2 serum–treated HSV-2–infected Vero cells. Results were compared with B cells (FcγR negative) by 1-way ANOVA. (**F**) Representative flow plots of Rhodamine123 expression in neutrophils or macrophages/monocytes cocultured with HSV-infected Vero cells treated with either control (black) or ΔgD-2 (red) serum. (**G**) Change in fluorescent intensity of DHR123 staining in isolated neutrophils or macrophages/monocytes in the ΔgD-2 immune serum–treated HSV-2 infected Vero cells compared with control serum–treated targets (*n* = 5). The difference in RNOS production was compared by 1-way ANOVA with Holm-Šidák’s multiple comparisons test (***P < 0.001, ****P < 0.0001).

Classic ADCC is mediated by the release of perforin and granzyme by effector immune cells, allowing granzyme to enter the cytosol of the target cell to initiate cell killing. To determine if this mechanism contributed to the cytolysis of HSV-infected targets, immune cells isolated from either the spleen or bone marrow of WT mice were incubated with HSV-infected Vero cells that had been pretreated with pooled immune serum from ΔgD-2 or control vaccinated mice. After a 4-hour incubation, the cells were stained and analyzed by flow for intracellular granzyme and perforin (protein transport inhibitor cocktail was added at 2 hours). Alternatively, an antibody to CD107a was added prior to coculturing immune cells with opsonized targets to capture its expression. There was no significant increase in intracellular granzyme B or perforin in any cell when cocultured with targets cells that had been pretreated with ΔgD-2 immune serum after subtracting the background production when the cells were cultured with targets pretreated with control serum. (Figure 3, A and B, Supplemental Figure 3A, and Supplemental Figure 4, A and B). There was also no significant increase in cell surface CD107a expression in any of the immune cell subpopulations (Figure 3C, Supplemental Figure 3B, and Supplemental Figure 4C). We used B cells as the control for comparison since they do not express FcγRIV. These findings indicate that none of the immune cells are secreting canonical ADCC cytotoxic granules.

NFAT activation, the biomarker in the mFcγRIV ADCC reporter assay, may be associated with the production of RNOS, which could contribute to target cell killing (51, 52). To assess this, bone marrow–derived cells were stained with dihydrorhodamine 123 (DHR123) prior to incubation with antibody-opsonized HSV-infected target cells. All bone marrow–derived cells generated RNOS when cocultured with ΔgD-2 incubated target cells (Figure 3D). There was a significant increase in the fluorescent intensity of rhodamine 123, a reporter of RNOS production when DHR123 is oxidized, in macrophages (*P* < 0.001) and neutrophils (*P* < 0.0001) when cocultured with targets preincubated with ΔgD-2 immune serum compared to B cells (Figure 3E). To validate these findings, neutrophils and macrophages/monocytes were isolated by negative selection to improve cell yields, stained with DHR123, and cultured with target cells incubated with either control or ΔgD-2 immune serum (Figure 3F). The isolated neutrophil population produced significantly more RNOS when targets were incubated with ΔgD-2 immune serum compared with control serum (*P* < 0.0001) and compared with myeloid cells (*P* < 0.0001) (Figure 3G). Moreover, the isolated macrophages/monocytes showed no difference in RNOS production when cultured with HSV-infected cells treated with ΔgD-2 versus control immune serum.

### Neutrophils trigger antibody-dependent killing of HSV-infected targets by trogocytosis.

**Figure 4:**
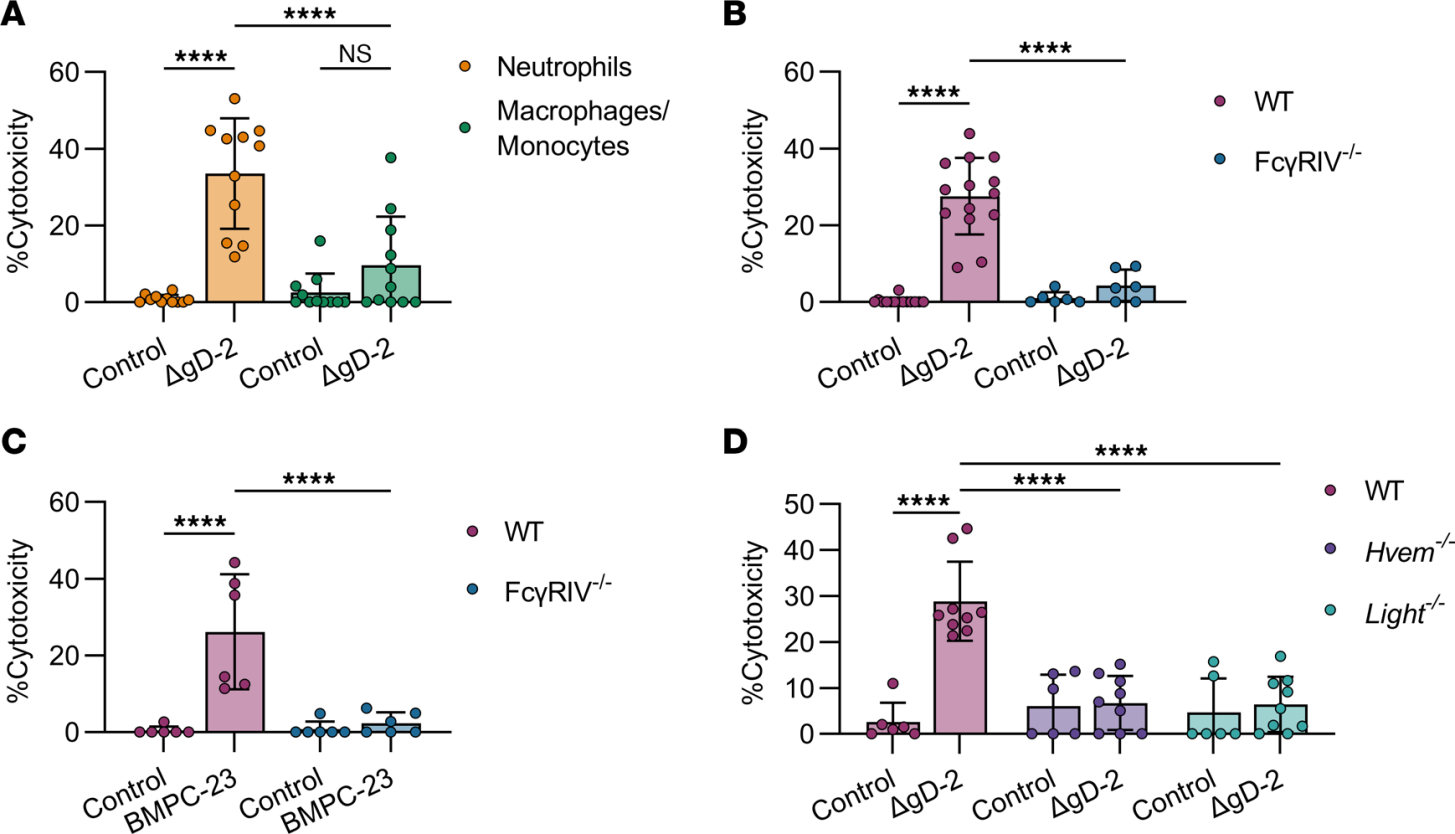
Mouse neutrophils require FcγRIV, HVEM, and LIGHT to mediate antibody-mediated cytolysis of HSV-infected target cells. (**A**) HSV-infected Vero cells were preincubated with ΔgD-2 or control immune serum (1:5 dilution) for 15 minutes and then cocultured with either isolated neutrophils or macrophages/monocytes for 2 or 4 hours, respectively (*n* = 11). Cytotoxicity was measured by the quantification of calcein released into the culture supernatant and is expressed as a percentage of maximum calcein release. (**B**) Neutrophils were isolated from WT or *Fc*γ*RIV^–/–^* mice and cocultured with target cells coated with ΔgD-2 or control immune serum for 2 hours or (**C**) BMPC-23 or an anti-mouse IgG2a isotype (10 μg) (control) for 4 hours (*n* = 6–14). (**D**) Neutrophils were isolated from WT, *Hvem^–/–^*, or *Light^–/–^* mice and cocultured with ΔgD-2 or control immune serum–pretreated HSV-infected targets (*n* = 6–9). Differences in cytotoxicity were compared by 1-way ANOVA with Holm-Šidák’s multiple comparisons tests (***P < 0.001, ****P < 0.0001).

The observation that neutrophils produced significant amounts of RNOS and also express mFcγRIV, HVEM, and LIGHT suggested that they may play a predominant role in mediating the ADCK of HSV-infected targets. To directly measure target cell killing, we used the fluorescent dye calcein-AM, which can be quantified fluorometrically, as it is released into the culture medium by dying cells. Isolated neutrophils and macrophages/monocytes from WT mice were cocultured with calcein-AM loaded HSV-2 infected Vero cells that had been preincubated with ΔgD-2 or control immune serum. Significantly more calcein was released when neutrophils were cocultured with ΔgD-2 compared with control immune serum treated targets (*P* < 0.0001) (Figure 4A). Significantly more calcein was also released by isolated neutrophils compared with isolated monocytes/macrophages (*P* < 0.0001). In addition, isolated macrophages/monocytes showed no significant increase in cytotoxicity when cocultured with target cells treated with ΔgD-2 immune serum compared with control serum. To validate that the observed neutrophil-mediated cytotoxicity is dependent on FcγRIV, neutrophils from *Fc*γ*RIV^–/–^* mice were cultured with either ΔgD-2 or control immune serum–treated target cells. Consistent with Figure 4A, neutrophils isolated from WT mice induced greater cytotoxicity with ΔgD-2 immune serum compared with control serum (*P* < 0.0001) but, the response was abrogated in neutrophils isolated from the *Fc*γ*RIV^–/–^* mice (*P* < 0.0001) (Figure 4B). Similar results were obtained when the HSV-infected targets were pretreated with BMPC-23, an HSV-specific mAb isolated from ΔgD-2 immunized mice and previously shown to provide protection through ADCC (*P* < 0.001) (53) (Figure 4C). However, little or no calcein release was detected when neutrophils isolated from *Hvem^–/–^* (*P* < 0.0001) or *Light^–/–^* mice (*P* < 0.001) were cocultured with ΔgD-2 immune serum opsonized HSV-infected target cells (Figure 4D).

**Figure 5:**
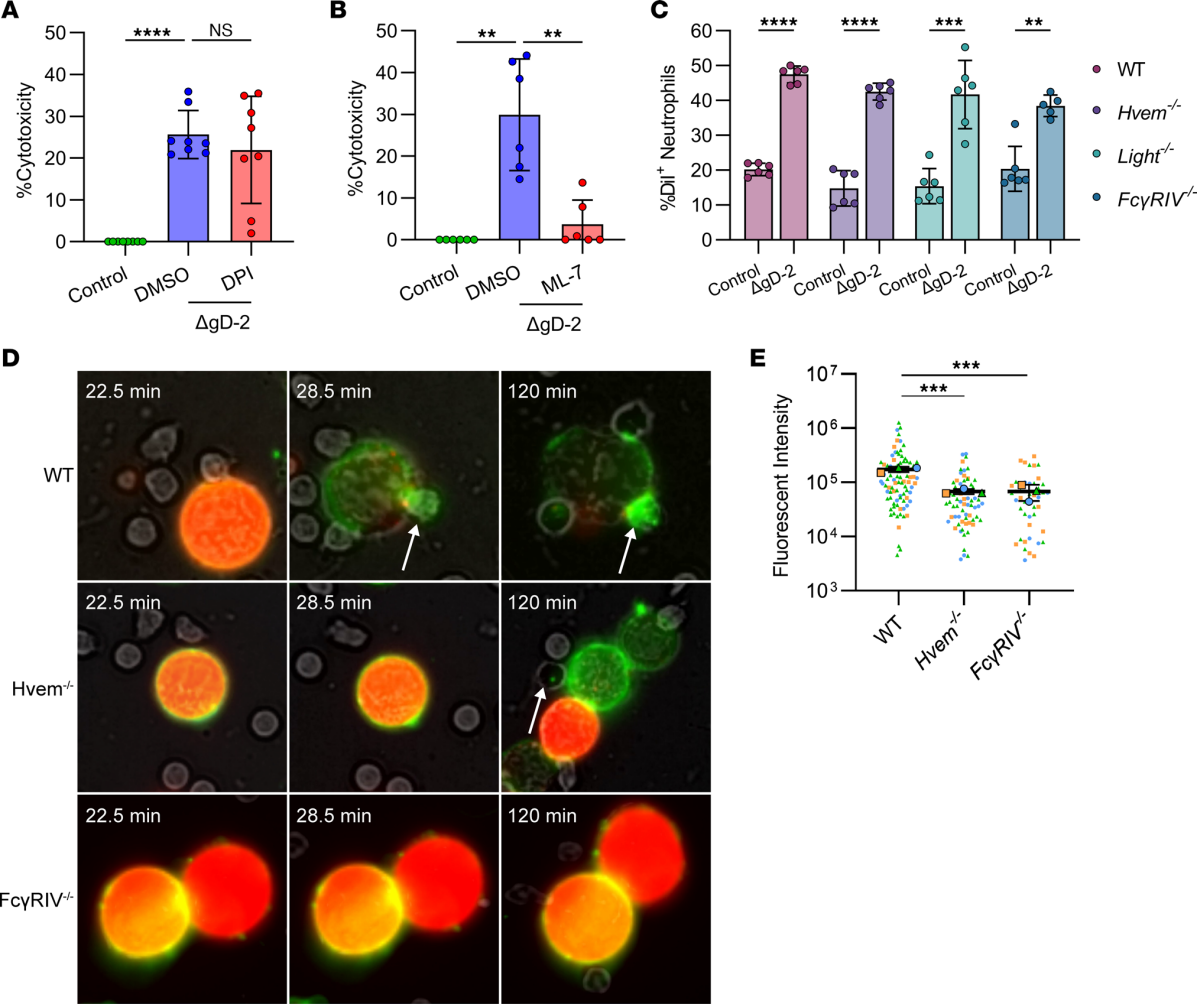
Killing of HSV-infected cells is mediated by trogocytosis and promoted by HVEM and LIGHT. WT mouse neutrophils were preincubated with (**A**) DPI, (**B**) ML-7, or DMSO buffer as a control and cocultured with ΔgD-2 or control immune serum pretreated HSV-infected, calcein-labeled Vero target cells. The percent cytotoxicity was quantified by measuring cell-free calcein in ΔgD-2 minus control serum treated targets (*n* = 6–8). (**C**) HSV-infected Vero cells were labeled with Vybrant DiI dye, incubated with ΔgD-2 or control immune serum, and neutrophils isolated from WT, *Hvem^–/–^*, or *Light^–/–^* mice. The percentage of DiI uptake was measured by flow cytometry on live neutrophils incubated with ΔgD-2 versus control immune serum (*n* = 6). (**D**) DiI-labeled (green) and calcein-AM–labeled (red) HSV-infected Vero cells were incubated with ΔgD-2 immune serum with neutrophils isolated from WT, *Hvem^–/–^*, or *Fc*γ*RIV^–/–^* neutrophils. Cytotoxicity was imaged over a 2.5-hour incubation with the Operetta CLS imaging system with images collected at 20 × magnification over 3 fields (*n* = 3). Representative images are shown at 3 timepoints: 22.5, 28.5, and 120 minutes after the start of imaging. (**E**) Fluorescent intensity of DiI^+^ neutrophils was measured with the Spot Intensity Analysis plugin using ImageJ across 3 fields (*n* = 3; orange, blue, and green) for WT, *Hvem^–/–^*, and *Fc*γ*RIV^–/–^*. Fluorescent intensity is reported for individual DiI^+^ neutrophils and the mean intensity in each field. Cytotoxicity and %DiI^+^ neutrophil were compared by repeated measures or 1-way ANOVA, respectively with Holm-Šidák’s multiple comparisons test; Fluorescent Intensity was compared by 1-way ANOVA with Holm-Šidák’s multiple comparisons test against the means of each field (**P < 0.01, ***P < 0.001, ****P < 0.0001).

Neutrophils mediate ADCC against cancer cells by trogocytosis, an FcγR-dependent process in which the neutrophils cluster around an antibody-opsonized target cell. This clustering results in membrane lipid translocation, disruption of the target cell membrane, and target cell death, which may be associated with and/or augmented by the release of RNOS. To assess whether trogocytosis contributes to neutrophil mediated ADCK of HSV-infected targets and whether RNOS is involved in this response, we tested the inhibitory impact of 2 drugs on antibody-dependent killing; ML-7, an inhibitor of myosin light chain kinase (MLCK) and diphenyliodonium chloride (DPI), which blocks intracellular NADPH oxidase, produced RNOS intermediates. Treatment of WT neutrophils with DPI had no inhibitory effect on cytotoxicity (Figure 5A), whereas treatment with ML-7 resulted in a significant reduction in target cell death as measured by calcein release (*P* < 0.01) (Figure 5B).

To determine how HVEM and LIGHT contribute to this process, we incubated neutrophils isolated from WT or KO mice with immune serum–opsonized HSV-infected targets that had been labeled with the membrane lipophilic dye, Vybrant DiI. Flow cytometry analysis showed a significant increase in the percentage of neutrophils that stained positive for DiI after pretreatment with ΔgD-2 versus control immune serum (*P* < 0.0001), a finding consistent with neutrophils acquiring membrane fragments from the targets (Figure 5C). There was also a similar response in neutrophils isolated from *Hvem*^–/–^, *Light*^–/–^, and *Fc*γ*RIV^–/–^* mice with only a small decrease in the percentage of DiI^+^ neutrophils comparing cells from the WT and KO mice. This did not match the magnitude of differences in cytotoxicity. To address this discrepancy, we performed live cell imaging, in which antibody-opsonized targets were loaded with calcein-AM to monitor cell death and membranes stained with DiI. Robust sharing of DiI membrane dye with neutrophils and subsequent target cell death (loss of intracellular calcein) was observed when targets were cocultured with WT neutrophils (Figure 5D and Supplemental Videos 1–3). The response was delayed and of lesser magnitude when targets were cocultured with neutrophils isolated from *Hvem^–/–^* mice and no loss of calcein dye was observed with FcγRIV-KO mice. Quantification of DiI^+^ staining in the neutrophils showed significantly less dye expression in neutrophils isolated from *Hvem^–/–^* (*P* < 0.001) and *Fc*γ*RIV^–/–^* compared with the WT mice (*P* < 0.001) (Figure 5E). These findings suggest that, although neutrophils from *Hvem^–/–^* mice extract membrane patches from target cells pretreated with ΔgD-2 (but not control) immune serum, the magnitude is less and they are impaired in mediating cytolysis.

### HVEM signaling promotes phosphorylation of BTK, PLCγ2, and MLCK.

**Figure 6:**
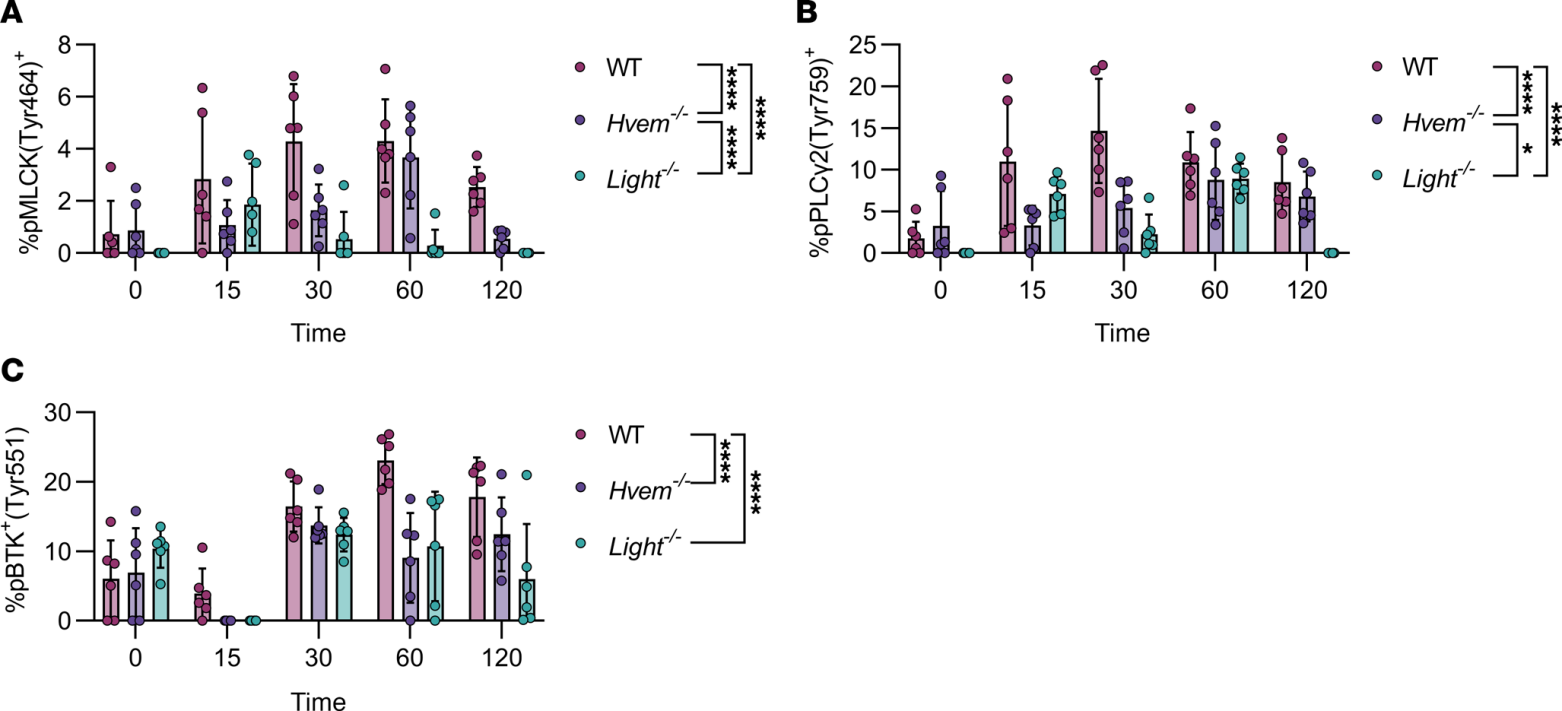
HVEM signaling promotes phosphorylation of the MLCK signaling pathway. (**A**–**C**) HSV-infected Vero cells were preincubated with ΔgD-2 immune serum (1:5 dilution in DMEM) before addition of WT, *Hvem^–/–^*, or *Light^–/–^* neutrophils (1:50 target:effector ratio) (*n* = 6 mice). Cells were harvested at indicated time points and phosphorylation of MLCK, PLCγ2, and BTK were determined by flow cytometry. Phosphorylation kinetics were compared between strains by a Gaussian nonlinear regression model with an extra sum-of-squares F test (*P < 0.05, ****P < 0.0001).

The decrease and delay in the amount of membrane captured and the impaired killing response suggest that HVEM signaling may contribute to the activation and phosphorylation of MLCK. To test this, we monitored the kinetics of MLCK phosphorylation in our cytolytic assay. HSV-infected Vero cells were incubated with ΔgD-2 immune serum and then cocultured with WT, *Hvem^–/–^*, or *Light^–/–^* neutrophils and, at the indicated times, the cells were harvested and stained for phosphorylated MLCK and analyzed by flow cytometry (Figure 6A and Supplemental Figure 5). Phosphorylation of MLCK occurred rapidly in neutrophils isolated from WT mice, peaking at approximately 30 minutes, with a sustained signal for at least 2 hours. In contrast, the response was delayed, of decreased magnitude, and was not sustained in neutrophils isolated from *Hvem^–/–^* mice and more so in neutrophils isolated from *Light^–/–^* mice (*P* < 0.0001). A similar difference was observed comparing the phosphorylation kinetics of PLCγ2, which is activated upstream of MLCK (14) (*P* < 0.0001) (Figure 6B and Supplemental Figure 6). Further dysregulation was observed in the phosphorylation of BTK, which is activated upstream of PLCγ2 and has potential binding sites for TRAFs recruited by HVEM activation (54, 55) (*P* < 0.0001) (Figure 6C and Supplemental Figure 7). The transient decrease in pBTK expression in *Hvem^–/–^* and *Light^–/–^* neutrophils at 15 minutes may represent in inability to sustain BTK phosphorylation during early neutrophil activation before the initiation of trogocytosis, which has been shown not to start until after approximately 15 minutes (56). The subsequent recovery may reflect a compensatory response perhaps involving other pathways such as TLR activation (57).

### HVEM expression on neutrophils is required for in vivo protection against lethal HSV challenge.

**Figure 7:**
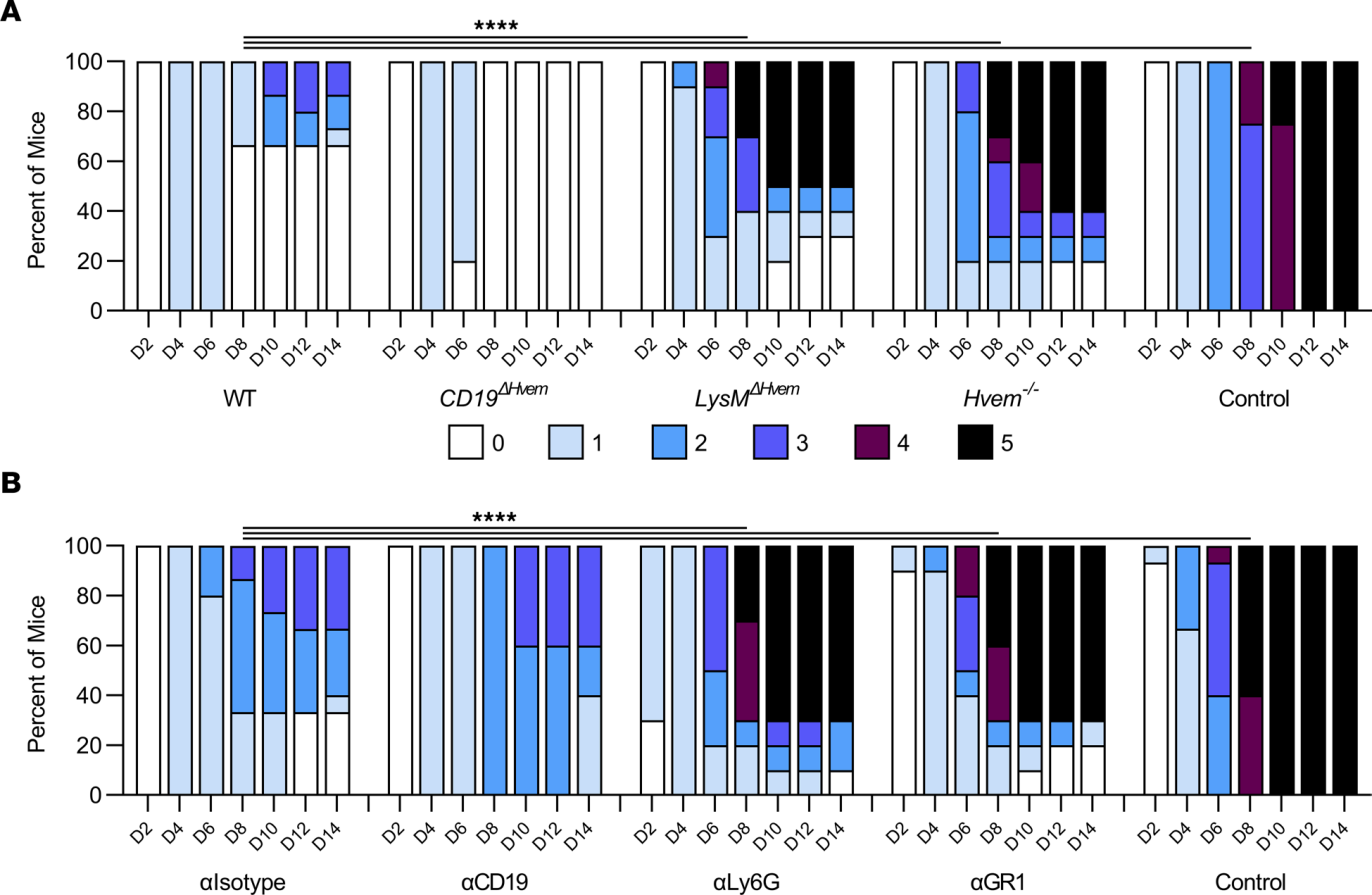
HVEM expression by neutrophils is required for passive immune protection against lethal viral challenge. (**A**) WT and the indicated cre/lox mice received immune serum from ΔgD-2–vaccinated mice containing 750 μg of IgG 1 day prior to challenge on the skin with a lethal dose of HSV-2 SD90. An additional group of WT mice received control immune serum. Mice were scored daily and disease scores compared with the WT mice that received control immune serum by 2-way ANOVA with *Šidák*’s multiple comparisons test (***P < 0.001 and ****P < 0.0001). (**B**) Immune cell subpopulations were depleted with neutralizing or isotype control mAbs and then the mice were intraperitoneally administered ΔgD-2 immune serum as in (**A**) and challenged on the skin with a lethal dose of HSV-2 24 hours later. An additional control for infection included mice that received control immune serum. The protective efficacy of the ΔgD-2 immune serum was compared for each mAb depletion group to mice that received isotype-control antibodies (2-way ANOVA with *Šidák*’s multiple comparisons tests, ****P < 0.0001, *n* = 10 mice per group from 2 independent experiments).

To determine if the in vitro findings that HVEM expression by neutrophils is important for antibody-dependent passive immune protection, we took advantage of the Cre/Lox system to target deletion of HVEM on specific immune cell populations. *Hvem^fl^* mice were crossed with *LysM^Cre^* (high efficiency deletion in granulocytes and less DC subsets) and *CD19^Cre^* (CD19^+^ B cells). Mice were crossed over 3–4 generations to produce mice hemizygous for the *Cre* alleles and homozygous for *Flox* alleles. Mice were genotyped to confirm transgene insertion and immunophenotyped by flow cytometry to confirm the specificity of HVEM deletion (Supplemental Figure 8). Passive transfer of 750 μg ADCC-replete ΔgD-2 immune serum into naive WT and *CD19**^ΔHVEM^*** provided complete protection, whereas protection was reduced when immune serum was transferred into *LysM**^ΔHVEM^*** and, as a control, *Hvem^–/–^* mice (*P* < 0.0001) (Figure 7A).

As a complementary approach, we treated WT mice with neutralizing antibodies to deplete different immune cell populations before passive transfer. CD19^+^ B cells were depleted by 2 0.15 mg injections of anti-CD19, 2- and 1-days before challenge. Neutrophils were depleted by 0.15 mg and 0.30 mg injections of anti-Ly6G, 2- and 1-days before challenge, respectively. Myeloid lineage cells and granulocytes were depleted by 0.15 mg and 0.30 mg injections of anti-GR1, 2- and 1-days before challenge, respectively. Negative control mice were injected 2- and 1-days before challenge with a cocktail of 0.15 mg of Ig2a and IgG2b isotype controls. Depletion of the subpopulations was confirmed by flow cytometry (Supplemental Figure 9). Passive protection of ΔgD-2–vaccinated mouse immune serum was preserved in mice treated with anti-CD19 or isotype control mAbs, but, consistent with the cre/lox results, was significantly reduced when Ly6G^+^ neutrophils or GR1^+^ myeloid lineages and granulocytes were depleted (*P* < 0.0001) (Figure 7B). The small number of mice that survived the α-GR1 treatment could reflect incomplete ablation of myeloid cells.

### HVEM-LIGHT facilitate human neutrophil mediated FcγR antibody-dependent trogocytosis.

**Figure 8:**
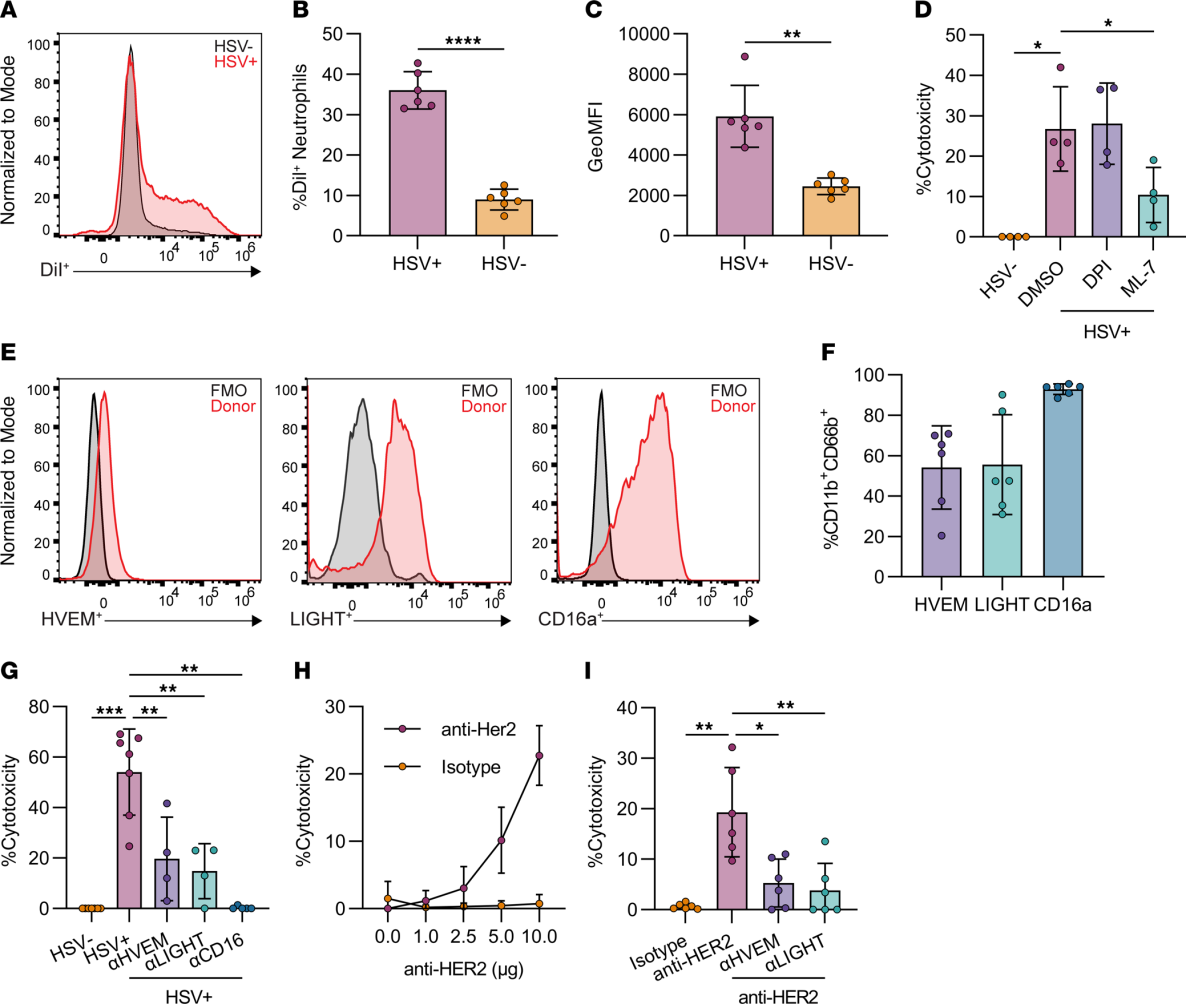
HVEM and LIGHT contribute to antibody-dependent trogocytosis by human neutrophils against HSV-infected targets and anti-HER2 opsonized human breast cancer cells. Following removal of PBMCs and RBC lysis from buffy coats, neutrophils (*n* = 6 donors) were cocultured with DiI-labeled HSV-infected Vero cells pretreated with serum from HSV+ donor containing ADCC-mediating antibodies or HSV– donors. (**A**) Representative flow plots of target cell DiI cell membrane detection in CD11b^+^CD66b^+^ neutrophils. (**B**) Percentage or (**C**) fluorescent intensity of neutrophils positive for target cell membrane detection with either HSV+ or HSV– serum. %DiI^+^ neutrophils and GeoMFI were compared by unpaired *t* test (**P < 0.01, ****P < 0.0001). (**D**) Neutrophils were pretreated with DPI, ML-7, or DMSO control before incubation with human serum opsonized HSV infected cell targets in the calcein release assay. Cell-free calcein was measured and converted to percentage cytotoxicity. (**E**) Representative flow plots of CD11b^+^CD66b^+^ neutrophils for expression HVEM, LIGHT, and CD16a in either FMO (black) or a donor sample (red). (**F**) Expression of HVEM, LIGHT, and CD16a as measured by flow cytometry from human donor neutrophils (*n* = 6). (**G**) Enriched neutrophils (*n* = 6 different donors) were incubated with antibodies blocking HVEM, LIGHT, or CD16a prior to incubation with human serum-treated (HSV+ or HSV– donors) HSV-infected calcein-labeled Vero cells. (**H**) Human donor neutrophils (*n* = 3) were incubated with calcein-labeled SKBR3 cells opsonized with increasing doses of a Trastuzumab biosimilar anti-HER2 mAb or an isotype control for 4 hours in the calcein release assay. (**I**) Calcein-labeled SKBR3 cells were treated with 10 μg of the anti-HER2 mAb and then cocultured with human neutrophils that had been pretreated with blocking antibodies against HVEM, LIGHT, or an isotype control and cytotoxicity monitored by measuring calcein release and presented as percentage cytotoxicity. Cytotoxicity was compared by 1 way ANOVA with Holm-Šidák’s multiple comparisons test (*P < 0.05, **P < 0.01, ***P < 0.001).

To determine whether the pathway of antibody-dependent granulocyte trogocytosis identified in the mouse model translates to humans, we isolated neutrophils from buffy coats and conducted assays with HSV-infected Vero cells treated with immune serum from HSV seropositive (*n* = 2) and HSV-seronegative (*n* = 2) donors (Supplemental Figure 10). The fold activation of human FcγRIIIA (CD16a) in the ADCC biomarker assay, was 26–28.5-fold in the seropositive and 2.4 in the seronegative serum (Supplemental Figure 11). We first confirmed that human neutrophils were acting in a similar mechanism to those observed in the mice. There was a significant increase in the percentage of human neutrophils taking up the lipophilic membrane dye when cultured with DiI-labeled target cells pretreated with HSV+ versus HSV– serum (*P* < 0.0001) (Figure 8, A and B). Additionally, the amount of target cell membrane detected in the neutrophils was higher when cultured with HSV+ versus HSV– serum-treated targets (*P* < 0.01) (Figure 8C). To directly assess killing, neutrophils were cultured with calcein-labeled HSV-infected target cells pretreated with human HSV+ or HSV– serum. The neutrophils induced significantly greater killing of the HSV+ versus HSV– treated target cells (*P* < 0.05) and the addition of ML-7 but not DPI reduced the response (*P* < 0.05) (Figure 8D).

We next tested whether the neutrophil-mediated antibody-dependent trogocytosis of HSV-infected targets (evidenced by DiI uptake and MLCK inhibition) was also associated with HVEM-LIGHT signaling. We isolated neutrophils from 6 different donors and assayed for expression of the proteins by flow cytometry. The expression of HVEM (63.25% [33.23%–70.21%]) and LIGHT (47.42% [34.23%–84.04%]) varied between donors, whereas CD16a was uniformly expressed by almost all live neutrophils (Figure 8, E and F). The neutrophils induced greater cytotoxicity when targets were treated with HSV+ versus HSV– immune serum (*P* < 0.001) and the addition of blocking antibodies targeting HVEM (*P* < 0.01), LIGHT (*P* < 0.01), or CD16a (*P* < 0.01) significantly reduced this cytolytic activity (Figure 8G).

To assess whether HVEM-LIGHT contributed to neutrophil mediated trogocytosis in other non-HSV contexts, we performed cytotoxicity (calcein) assays using anti-HER2 mAb-coated human breast cancer cells (SKBR3). Neutrophils have been previously shown to kill anti-HER2–treated SKBR3 cells by trogocytosis (14). Neutrophils were incubated with calcein-labeled SKBR3 cells coated with increasing amounts of a Trastuzumab Biosimilar anti-HER2 antibody or a hIgG1 isotype control. A dose-dependent cytolytic effect was observed with target cells treated with increasing concentrations of the anti-HER2 antibody (*P* < 0.001 compared with hIgG1 isotype treated targets at 10 μg) (Figure 8, H and I). Using this dose, we found that the addition of blocking antibodies against HVEM (*P* < 0.05) and LIGHT (*P* < 0.01) significantly inhibited this cytolytic response.

## Discussion

The term trogocytosis was initially coined to describe the process by which amoebae kill cells, which was later found to play roles in cell-cell communication; for example, the transfer of MHC molecules from antigen presenting cells to T cells at the immunologic synapse (58, 59). It was subsequently shown that neutrophils utilize trogocytosis to kill parasites such as *Trichomonas vaginalis* (60), and, more recently, to mediate antibody-dependent killing of cancer cells (14). Our findings expand on these trogocytosis-dependent processes and begin to define the mechanistic basis of neutrophil-mediated, antibody- and FcγR-dependent killing of HSV-infected cells. The neutrophils kill the antibody-opsonized targets through a process involving cytoskeletal contractions rather than via the canonical mechanisms involving cytotoxic granules, phagocytosis, or release of neutrophil extracellular traps (NETs). This ADCK process depends not only on expression of FcγRs (mouse FcγRIV or human FcγRIIIA) but is potentiated by HVEM-LIGHT signaling. The role of HVEM-LIGHT in augmenting antibody-mediated trogocytosis is not restricted to HSV but was also observed using anti-HER2 mAb and breast cancer cells.

The evidence that the HVEM-LIGHT pathway provides a second signal and augments ADCK mechanisms initially came from our studies with a gD-null viral vaccine candidate. The vaccine, ΔgD-2, elicits high-titer polyfunctional antibodies that have little complement-independent neutralizing activity, but activate FcγRs and passively protect WT but not FcγRIV-KO mice, indicating that protection is primarily mediated by an FcγRIV-activating pathway. We subsequently showed and confirmed here that passive protection was also significantly reduced when the immune serum was transferred into *Hvem*^–/–^ or *Light*^–/–^ mice. Using cell-type–specific conditional knockouts or isolating specific subpopulations from the *Hvem*^–/–^ or *Light*^–/–^ mice, we now show that neutrophils play the dominant role in mediating this protective response. The in vitro cytolytic activity was significantly reduced if neutrophils were isolated from HVEM- or LIGHT-KO mice or if human or mouse neutrophils were treated with anti-HVEM or anti-LIGHT–blocking antibodies. The contribution of HVEM signaling to neutrophil-mediated killing was further supported by the in vivo findings that passive protection was lost when immune serum from ΔgD-2–vaccinated WT mice was transferred into mice lacking HVEM on their neutrophils (*LysM**^ΔHVEM^*** mice).

When HVEM engages LIGHT, it recruits TRAF proteins to initiate downstream signaling responses that culminate in activation of NF-kB and JNK/AP-1 pathways (42, 61). We previously showed a role for HVEM signaling in NF-kB activation using an ADCC reporter assay. Specifically, the addition of HVEM-blocking antibodies or soluble gD protein (which binds HVEM thereby serving as an immune evasion strategy) reduced NFAT activation when Jurkat cells engineered to express either mouse FcγRIV or human FcγRIIIa and an NFAT-luciferase reporter were cocultured with ADCC-mediating antibody-opsonized HSV-infected targets (27). The current studies demonstrate that HVEM-LIGHT signaling provides a second signal and promotes kinase responses upstream of NFAT activation, including the phosphorylation of BTK, PLC-γ, and MLCK. Activation of FcγRs has been previously shown to recruit PLC-γ to the cell membrane, where it is phosphorylated by BTK, triggering downstream calcium signaling responses including phosphorylation of MLCK (14, 62). Activation of MLCK, in turn, triggers the cytoskeletal contractions mediating trogocytosis (14). Using mouse or human neutrophils, we demonstrate that this signaling response to FcγR crosslinking is attenuated if HVEM or LIGHT are absent (e.g., using neutrophils isolated from knockout mice) or if HVEM-LIGHT signaling is inhibited by the addition of blocking antibodies (in studies with human neutrophils). In monocytes, it has been previously shown that binding of LIGHT to HVEM recruits TRAF adaptor proteins to the intracellular domain of HVEM, which promotes phosphorylation of PLC-γ1 (63). Our data indicate a similar mechanism in neutrophils where LIGHT binding to HVEM promotes the phosphorylation of PLC-γ2, which is the preferential isoform expressed by neutrophils (64).

While the results reported here demonstrate a dominant role for neutrophils in mediating killing of antibody-opsonized HSV-infected targets via a trogocytosis-like pathway, they do not preclude a role for other immune effector cells or other killing mechanisms. For example, we observed increased production of RNOS when monocytes, macrophages, and neutrophils were cocultured with antibody-opsonized HSV-infected targets. While pharmacologic inhibition of RNOS production did not lead to a significant loss in cytolytic activity in vitro, RNOS may contribute to clearance of virally infected cells in vivo. The varied RNOS production and trending increase in cytotoxicity in macrophages/monocytes may be representative of antibody-dependent cellular phagocytosis (ADCP), a FcγR-mediated killing observed with the ΔgD-2 vaccine (23). RNOS facilitate killing following phagocytosis by macrophages, a process that would result in engulfment of target cell calcein, rather than release from lysed cells (65). We also cannot exclude a role for NK cells in mediating ADCC, as mouse NK cells, unlike human NK cells, express only low levels of FcγRIV, the dominant mediator of ADCC in mice. The in vitro observations of neutrophil killing may not fully recapitulate other HVEM linked interactions that occur in vivo. For example, trafficking of neutrophils to sites of HSV infection requires cytokines produced by other cells whose function may also be enhanced by HVEM signaling (66, 67).

A dominant role for neutrophils in protecting against HSV is consistent with earlier studies as well as clinical experience. For example, killing of infected cells by neutrophils was only observed in the presence of HSV-immune serum and cells from patients with chronic granulomatous disease (CGD) were as effective as cells from healthy control cells in the in vitro assays, indicating that killing was independent of the respiratory burst, although the mechanism was not further addressed (68). Consistent with this in vitro observation, patients with CGD are not at increased risk for HSV. In contrast, neutropenia is associated with an increased risk for primary and recurrent HSV disease and HSV viremia, and antiviral prophylaxis with acyclovir or valacyclovir is recommended by the Infectious Disease Society of America and American Society of Clinical Oncology for high-risk neutropenic patients, including stem cell transplant recipients, patients with acute myeloid leukemia, and patients with myelodysplastic syndromes (69–72).

The ability of the HVEM-LIGHT pathway to augment the ADCC pathways is not restricted to HSV or, specifically, to neutrophil-mediated trogocytosis. For example, we previously demonstrated that the addition of anti-HVEM–blocking antibodies or soluble gD protein to the ADCC reporter assay inhibited the NFAT response when rituximab (anti-CD20) was cocultured with Raji B cells (27), and we now show a role for HVEM-LIGHT in neutrophil mediated killing of breast cancer cells by anti-HER2. While neutralizing antibodies have been presumed to be the primary correlate of immune protection against HSV and have dominated vaccine development, the need to elicit cytolytic antibodies that act through FcγR activation have been increasingly recognized, particularly for viruses that can evade neutralization by spreading directly through cell-cell contacts, including herpesviruses (HSV and CMV), HIV, RSV, and others (73). We speculate that the efficacy of ADCC-mediating antibodies to eliminate cells infected with these viruses is also enhanced by HVEM-LIGHT signaling whether the mechanism of killing involves trogocytosis or classic production of cytolytic granules. The findings here support future studies directed at developing strategies to promote the HVEM-LIGHT signaling pathway to enhance the cytolytic activity of monoclonal antibodies or vaccines targeting viral pathogens or tumor cells.

## Methods

### Sex as a biological variable.

Sex was not considered a biological variable in this study or in the data analysis. Both male and female mice and buffy coats from male and female humans were used in these experiments.

### Mice.

Age-matched male and female C57BL/6 mice were purchased from the Jackson Laboratory (JAX). *Hvem^–/–^* (Richard Longnecker, Northwestern University, Chicago, Illinois, USA) (74), *Light^–/–^*, *Btla^–/–^*, and *CD160^–/–^* (Carl Ware, Sanford Burnham Prebys Medical Discovery Institute, La Jolla, California, USA) (75–77) were bred at the Albert Einstein College of Medicine. *Fc*γ*RIV^–/–^* mice were obtained from Jeffery Ravetch (Rockefeller University, New York, New York, USA) (78). *LysM^Cre^* (Carl Ware), *CD19^Cre^* and *Hvem^fl^* mice (JAX) (79–81) were crossed for mice hemizygous for the *Cre* allele and homozygous for the *Flox* allele.

### Cell lines, viruses, and vaccines.

Vero (Green Monkey Kidney cell line, ATCC) and VD60 cells, a Vero cell line expressing HSV-1 glycoprotein D under the control of its own promoter (82), were grown in DMEM (Thermo-Fisher Scientific) supplemented with 10% FBS (Hyclone) and 1% penicillin-streptomycin (Thermo-Fisher Scientific). SKBR3 (HTB-30, ATCC) were cultured in DMEM supplemented with 20% FBS and 1% penicillin-streptomycin. The clinical isolates, HSV-2 (SD90) (83) (David Knipe, Harvard Medical School) and HSV-2 (4674) (23, 84) (Montefiore Clinical Virology Lab) were grown and viral titers determined by plaque assay. The ΔgD-2 vaccine strain was engineered and grown on VD60 cells as previously described and modified to remove the green fluorescent protein (85, 86). The virus was grown on VD60 cells and viral titers were determined by plaque assay on VD60 and Vero cells. No plaques were detected on Vero cells. The ADCC-mediating mouse mAb, BMPC-23, which was initially isolated from ΔgD-2 vaccinated mice, was cloned into a mouse IgG2c vector and purified as previously described (53).

### Immunization and infection of mice.

Male and female WT mice were vaccinated intramuscularly at 6-8 weeks old and then boosted two weeks later with 5 × 10^6^ plaque-forming units (pfu) of ΔgD-2-infected VD60 cell lysate or control uninfected VD60 cell lysate. One week after the boosting vaccine dose, mice were bled retro-orbitally and serum collected. Total serum IgG was calculated by ELISA (88-50400-22; Thermo-Fisher Scientific, Waltham, MA, USA). 750μg total IgG was injected intraperitoneally into naïve WT or knockout mice 1 day prior to challenge with a LD90 of HSV-2 (SD90) or HSV-2 (4674) as previously described (24). Mice were monitored daily for epithelial and neurological disease and scored as following: (a) erythema at inoculation site; (b) spread to distant site, zosteriform lesion; (c) edema, ulceration, epidermal spread, and/or paresis; (d) hind limb paralysis; (e) death. Mice with a score of 4 were euthanized and assigned a score of 5 the following day.

### Immune cell depletions.

Naïve WT mice were injected with 0.15mg of anti-Ly6G (clone 1A8; Bio X Cell), anti-GR1 (clone RB6-8C5; Bio X Cell), anti-CD19 (clone 1D3; Bio X Cell), or IgG2a isotype (clone 2A3; Bio X Cell) and IgG2b isotype (clone LTF-2; Bio X Cell) two days before challenge. Mice were injected again one day before challenge with 0.30mg of anti-Ly6G, anti-GR1, anti-CD19, IgG2a isotype, or IgG2b isotype along with ΔgD-2 immune serum. Mice were then challenged on the skin with a LD90 dose of HSV-2 (SD90). Depletion was confirmed by flow cytometry by retro-orbital bleed.

### ELISA.

HSV-binding IgG was measured by ELISA in serum collected one-week following the second vaccine dose. ELISA plates were coated with lysates of uninfected Vero cells or cells infected with HSV-2 (SD90) at a multiplicity of infection (MOI) of 0.1 pfu/cell for 24 hours. Serial dilutions of immunized mouse serum in duplicated were incubated with coated plates overnight at 4°C. Bound IgG was quantified using horseradish peroxidase (HRP)-conjugated secondary antibodies (Southern Biotech, Birmingham, AL, USA). Uninfected Vero cell lysate was subtracted from infected cell lysate to adjust for background. Results are presented as the optical density value measured at a 450nm filter.

### FcγR activation assay.

FcγR activation was assessed using Promega’s Mouse FcγRIV ADCC Bioassay or ADCC Reporter Bioassay, F Variant (Promega). Vero cells were infected at a MOI of 0.1 overnight with HSV-2 (SD90) and used as target cells. The targets were transferred to white, flat-bottom 96 well plates and incubated with heat-inactivated mouse or human serum diluted 1:5 in DMEM for 15 minutes at room temperature. FcγR reporter cells were incubated with target cells and serum for 6 hours at 37°C 5% CO_2_.

### Calcein release assay.

SKBR3 cells, HSV-2 infected or uninfected control targets were detached using CellStripper (Corning), pelleted by centrifugation, and resuspended at a concentration of 2 × 10^6^ cells/mL in RPMI 1640 supplemented with 1% heat-inactivated FBS and 1% penicillin-streptomycin. Cells were labeled with 2μM Calcein-AM (BioLegend, San Diego, CA, USA) for 30 minutes, washed in supplement RPMI 1640, and adjusted to a concentration of 2 × 10^6^ cells/mL. Effector cells included mouse neutrophils or monocytes/macrophages isolated from bone marrow harvested from both hind legs using the EasySep Mouse Neutrophil Enrichment Kit (StemCell Technologies, Vancouver, BC, Canada) or EasySep Mouse Monocyte Enrichment Kit (StemCell) per the manufacturer’s instructions or human granulocytes isolated from donor buffy coats (Gulf Coast Regional Blood Center, Houston, TX, USA) following removal of PBMCs by Percoll (Cytiva) gradient and ACK red blood cell lysis (Gibco). In indicated experiments, neutrophils/granulocytes were incubated with 25μM diphenyleneiodonium chloride (DPI), 5μM ML-7, or equivalent concentration of DMSO for 5 minutes before coculture.

The calcein release assay was performed in U-bottom 96-well plates (Corning, Corning, NY, USA). The target cells (1.0 × 10^4^ cells/well) were incubated for 15 minutes at room temperature with mouse immune serum (diluted 1:5 in DMEM), BMPC-23 (25μg/well), anti-Her2 (her2tra-mab1; InvivoGen) (0–10μg/well), or human serum from a repository of HSV seropositive or seronegative controls to opsonize the targets. The effector cells were added at a target:effector (T:E) ratio of 1:50 for neutrophils or 1:10 for macrophages/monocytes. Plates were incubated at 37°C 5% CO_2_ for 2 hours for mouse neutrophils, 4 hours for mouse monocytes, or 3 hours for human effectors. Control included 5 wells containing targets and effectors with isotype control or control mouse serum (background) and 5 wells containing 2% Triton X-100 (max release). Plates were centrifuged at 300 × g for 3 minutes, after which supernatant was transferred to a new 96-well plate. 75μL of the supernatant was transferred to a black 96-well flat bottom plate (Greiner Bio-One, Kresmünster, Austria). Fluorescent intensity was measured at an excitation of 488nm and an emission of 517nm using a SpectroMax M5 (Molecular Devices, San Jose, CA, USA). Cytotoxicity was calculated as [(sample release – background)/(max release – background)] × 100%.

### Flow cytometry.

Immune cells were dissociated from whole spleens or bone marrow into single cell suspension. Cells were incubated, at target:effector ratio of 1:10, with Vero cells previously infected overnight with HSV-2 at a MOI of 0.1 for 4 hours at 37°C 5% CO_2_. To assess degranulation, cells were stained with an antibody against CD107a (APC; clone 1D4B; SouthernBiotech) at the beginning of the incubation. Protein Transport Inhibitor Cocktail (Invitrogen, San Diego, CA, USA) were added to reactions for staining of intracellular cytokines when applicable. Surviving cells were transferred to V-bottom 96-well plates (Greiner Bio-One, Kresmünster, Austria). Cells were pre-stained for 10 minutes at room temperature with fixable viability dye Zombie NIR (BioLegend). Cells were washed and stained for 20 minutes on ice with antibodies against FcγRIV (BV650; clone 9E9; BioLegend), HVEM (PE; clone HMHV-1B18; BioLegend), LIGHT (AF647; PA5-104479; Invitrogen), granzyme B (AF488; clone QA18A28; BioLegend), perforin (AF647; clone S16009A; BioLegend), CD3 (AF700; clone 17A2; BioLegend), CD4 (BUV737; clone RM4-5; BD Biosciences), CD8 (BV510 or BV650; clone 53-6.7; BioLegend), Ly6G (AF594; clone 1A8; BioLegend), Ly6C (AF488 or AF700; clone HK1.4; BioLegend), CD11b (PE; clone M1/70; BioLegend), CD11c (PE-Cy7;clone N418; BioLegend), MHCII (BV421; clone M5/114.15.2; BioLegend), CD19 (APC-Fire810; clone 6D5; BioLegend), NK1.1 (BUV395; clone PK136; BD Biosciences), and F4/80 (PerCP-Cy5.5; clone BM8.1; Tonbo Biosciences). Samples were washed twice and resuspended in 2% paraformaldehyde in staining buffer for 15 minutes on ice. Samples were washed once, resuspended in staining buffer, and acquired on a Cytek Aurora flow cytometer. Immune cell populations were gated as followed: B Cells (CD19^+^CD3^–^), CD4^+^ T Cells (CD3^+^CD4^+^), CD8^+^ T Cells (CD3^+^CD8^+^), Macrophages (CD11b^+^F4/80^+^), Monocytes (CD11b^+^Ly6C^+^F4/80^–^), Neutrophils (CD11b^+^Ly6G^+^), NK Cells (NK1.1^+^CD4^–^CD8^–^), and Dendritic Cells (CD11c^+^MHCII^+^F4/80^+^).

For phosphorylated proteins, isolated neutrophils from WT or *Hvem^–/–^* mice were cocultured with HSV-infected Vero cells incubated in ΔgD-2 immune serum (diluted 1:5 in DMEM). Cells were removed at 0, 15, 30, 60, and 120 minutes and immediately spun at 300xg for 5 minutes. Cells were resuspended in CytoFix/CytoPerm Fixation/Permeabilization solution (BD) supplemented with protease and phosphatase inhibitor cocktails (Sigma) for 20 minutes on ice. Cells were washed in BD Perm/Wash buffer supplement with inhibitors and stained for 30 minutes on ice with antibodies against Ly6G (AF594; clone 1A8; BioLegend), CD11b (BV711; clone M1/70; BioLegend), pMLCK (PE; PA564763; Invitrogen), pPLCγ2 (AF647; clone 4NPRN4; Invitrogen) and pBTK (PE; clone A16064A; Invitrogen). Samples were washed twice in Perm/Wash supplemented buffer and twice in staining buffer. Cells were resuspended in staining buffer and acquired on a Cytek Aurora flow cytometer.

For human neutrophils, cells were isolated from buffy coats as previously described for single cell suspensions. Cells were either stained immediately for protein expression or cocultured with DiI-labeled HSV-infected Vero cells for 4 hours with either HSV+ or HSV– immune serum. Cells were pre-stained for 10 minutes with fixable viability dye Zombie NIR at room temperature. Cells were washed and stained on ice for 20 minutes with antibodies against CD66b (PE-Cy7; clone G10F5; BioLegend), CD11b (BV421; clone ICRF44; BioLegend), HVEM (APC; clone 122; BioLegend), LIGHT (PE; clone T5-39; BioLegend), and CD16a (PerCP; clone 41; Sino Biological). Samples were washed twice and resuspended in 2% paraformaldehyde in staining buffer for 15 minutes on ice. Samples were washed once, resuspended in staining buffer, and acquired on a Cytek Aurora flow cytometer. Neutrophils were gated on FSC^hi^SSC^hi^CD11b^+^CD66b^+^ cells.

### Reactive nitrogen and oxygen quantification.

Immune cells dissociated from whole spleen or bone marrow, or isolated monocytes or neutrophils were pretreated with Dihydrorhodamine 123 (DHR123) (ThermoFisher Scientific) for 15 minutes at room temperature. Cells were washed and then incubated with Vero cells previously infected overnight with HSV-2 at a MOI of 0.1 for 4 hours at 37°C 5% CO_2_. Target:effector ratios for whole spleen, bone marrow, or isolated macrophages/monocytes were 1:10, or 1:50 for isolated neutrophils. Cells were transferred to V-bottom 96-well plates and treated similarly for flow cytometry as previously mentioned.

### Genotyping.

3-week-old *CD19*^ΔHVEM^ and *LysM*^ΔHVEM^ mice were anesthetized with isoflurane prior to collection of tail biopsies. Biopsies were digested for DNA using the DNeasy Blood and Tissue Kit (Qiagen). DNA sequences for Cre and LoxP insertions were amplified by PCR using the following sequences: CD19-Common-Forward (AAT GTT GTG CTG CCA TGC CTC), CD19-WT-Reverse (GTC TGA AGC ATT CCA CCG GAA), CD19-Cre-Reverse (AAT GTT GTC GGA TAG TTT TTA CTG C), LysM-Common-Forward (CTT GGG CTG CCA GAA TTT CTC), LysM-WT-Reverse (TTA CAG TCG GCC AGG CTG AC), LysM-Cre-Reverse (CCC AGA AAT GCC AGA TTA CG), HVEM-Flox-Forward (ACC AAA TCA GAC CTG GGA AG), and HVEM-Flox-Reverse (TCC AGC TGT GTG ATC TAC CTC). Following amplification, PCR products were analyzed by 2% agarose gel electrophoresis and imaged on iBright 1500 Series Imaging System (ThermoFisher).

### Live cell imaging.

Vero cells were infected overnight at a MOI of 0.1 with HSV-2 SD90. Infected cells were dissociated with CellStripper (Corning) and resuspended at a concentration of 2 x 10^6^ cells/mL in DMEM. Target cell cytoplasm was labeled with 1μM Calcein-AM (BioLegend) and membranes stained with 5μM DiI (Invitrogen) at 37°C 5% CO_2_ for 10 minutes. After washing and resuspension in RPMI supplemented with 1% HI-FBS (Hycult), target cells were incubated with ΔgD-2 immune serum, prediluted 1:5 in DMEM, for 15 minutes. Target cells were incubated with mouse neutrophils at a T:E ratio of 1:5 for up to 2.5 hours in RPMI supplemented with 1% HI-FBS (Hycult). Imaging began 15 minutes after incubation of target cells with neutrophils. Images were collected at 20× from three independent fields, per strain, every 30 seconds in an Operetta CLS High-Content Analysis System (Revvity). Images were analyzed by Spot Intensity Analysis plugin using ImageJ.

### Statistics.

Flow cytometry data was analyzed by FlowJo software version 10.10.0 (Tree Star Inc, Ashland, OR, USA). Image analysis was done with ImageJ (NIH).

Statistical tests were performed with GraphPad PRISM version 9.5.0 (GraphPad Software). A *P* value of 0.05 was considered statistically significant. Disease scores were compared using a 2-way ANOVA with Šidák’s multiple comparison tests. Survival curves were compared using a Gehan-Breslow-Wilcoxon test. Flow cytometry protein and RNOS expression and cytotoxicity assays were compared using a 1-way ANOVA with multiple comparisons. Phosphorylation kinetics were compared by Gaussian nonlinear regression models with an extra sum-of-squares F test comparing unshared parameters between WT, *Hvem^–/–^*, and *Light^–/–^*. Flow cytometry staining for %DiI and GeoMFI of human neutrophils were compared by unpaired 2-tailed *t* tests (Figure 8).

### Study approval.

Animal studies were conducted in accordance with the Albert Einstein College of Medicine Institutional Animal Care and Use Committee protocol 0000-1287. Processing of human buffy coats was approved by the Albert Einstein College of Medicine Institutional Review Board under protocol 2015-5463.

### Data and materials availability.

All data relevant to the conclusions in the paper are accessible in the Supporting Data Values file or the Supplemental Materials. Materials described in the paper are available from the corresponding authors upon request.

## Author contributions

MSG, SCA, and BCH designed the experiments. MSG performed the experiments. Reagents were generated and supplied by MK and CFW. Funding was acquired by BCH and SCA. Original manuscript draft was written by MSG and BCH. The final draft was edited and reviewed by MK, SCA, and CFW.

## Conflict of interest

BCH and MK are coinventors on a patent (Patent no. US-20220162291-A1) assigned to Albert Einstein College of Medicine and Duke University regarding the HSV monoclonal antibody used in this study. BCH is a coinventor on a patent application (Patent no. US-20210361743-A1) assigned to Albert Einstein College of Medicine regarding HVEM signaling to promote ADCC.

## Funding support

This work is the result of NIH funding, in whole or in part, and is subject to the NIH Public Access Policy. Through acceptance of this federal funding, the NIH has been given a right to make the work publicly available in PubMed Central.

- NIH R01AI187024 (BCH and SCA).
- NIH R01AI177673 (BCH and MK).
- NIH P30AI124414 (BCH).
- Rowland and Sylvia Schaefer Family Foundation Inc. (BCH).

## Supplementary Material

Supplemental data

Unedited blot and gel images

Supplemental video 1

Supplemental video 2

Supplemental video 3

Supporting data values

## Version 1 06/04/2026

In-Press Preview

## Version 2 07/15/2026

Electronic publication
