# Supplementary material for: HVEM-LIGHT signaling promotes antibody-dependent neutrophil FcγR-mediated trogocytosis against herpes simplex virus infection
Source: J Clin Invest. 2026 Jun 4;136(14):e203771. doi: 10.1172/JCI203771 (PMC13367962; doi:10.1172/JCI203771)
Supplement: Supplemental data [file jci-136-203771-s316.pdf]

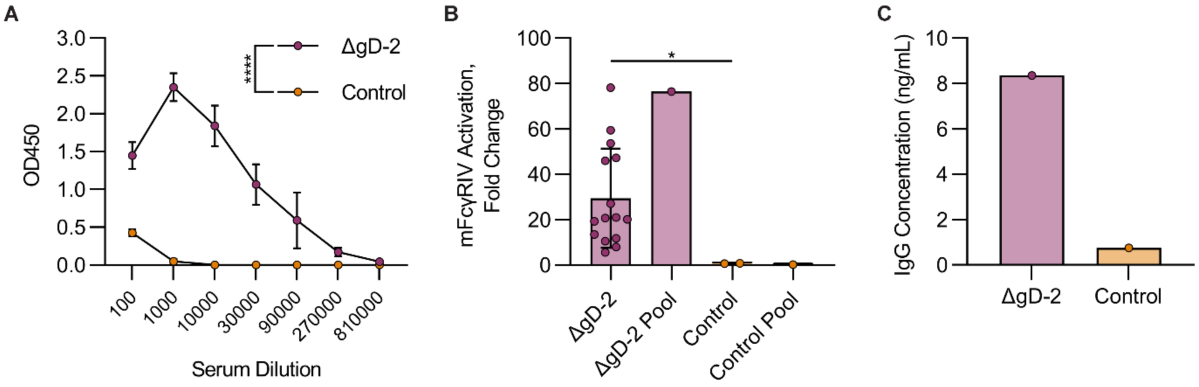

Figure S1. ΔgD-2 vaccination induces robust HSV-specific FcγRIV-activating antibody responses. WT C57BL/6J mice were vaccinated with $5x10^6$ pfu ΔgD-2 or control VD60 lysate two weeks apart. One-week post-boost, serum was collected and assessed for (A) total HSV-specific IgG, (B) FcγRIV activation using HSV-infected Vero cels (1:5 dilution), and (C) total IgG.

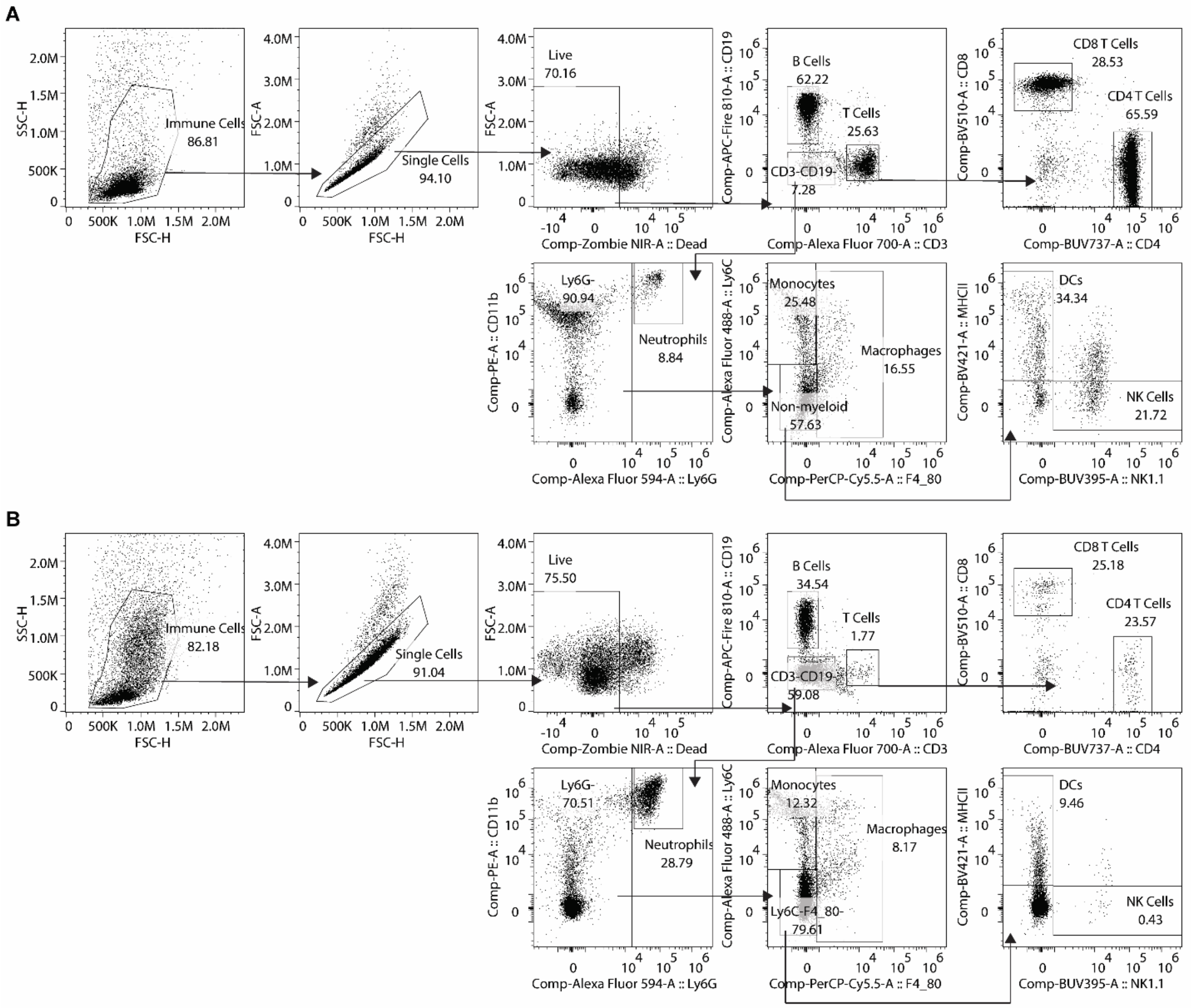

Figure S2. Gating strategy for immune cell populations for protein expression. WT mouse (A) spleens or (B) bone marrow were dissociated into single cell suspensions for flow cytometry analysis. Following gating by FSC/SSC, single cells were assessed for viability by Zombie NIR (BioLegend). Live cells were then divided into B cells ($CD19^{+}CD3^{-}$), $CD4^{+}$ T cells ($CD3^{+}CD4^{+}$), CD8 T cells ($CD3^{+}CD8^{+}$), Neutrophils ($CD19^{-}CD3^{-}Ly6G^{+}CD11b^{+}$), Monocytes ($CD19^{-}CD3^{-}Ly6G^{-}Ly6C^{+}$), Macrophages ($CD19^{-}CD3^{-}Ly6G^{-}F4/80^{+}Ly6C^{hi/lo}$), Dendritic cells ($CD19^{-}CD3^{-}Ly6G^{-}Ly6C^{-}F4/80^{-}MHCII^{+}$), and NK cells ($CD19^{-}CD3^{-}Ly6G^{-}Ly6C^{-}F4/80^{-}MHCII^{-}NK1.1^{+}$).

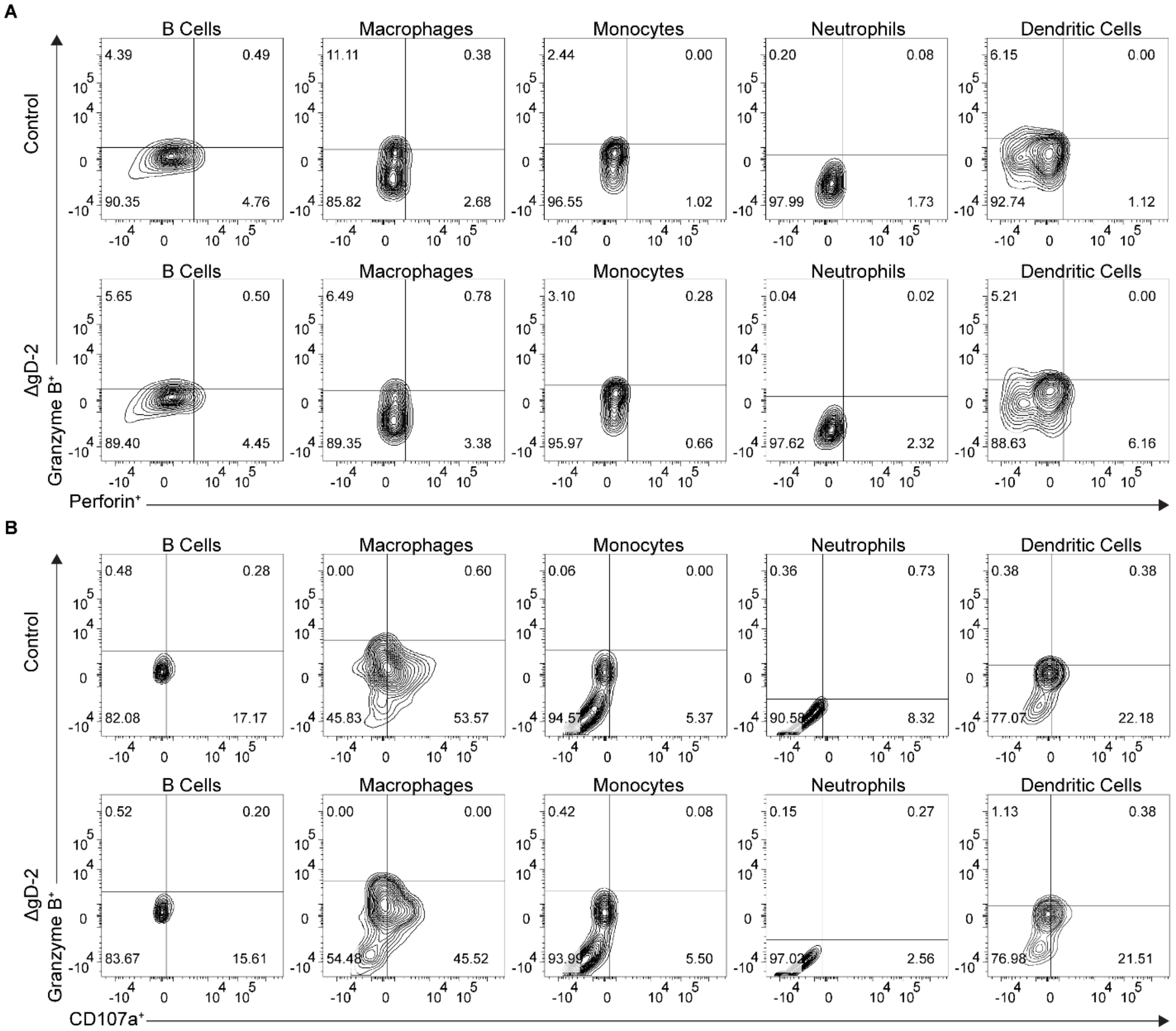

Figure S3: Representative flow plots for granzyme B, perforin, and CD107a staining. Immune cells isolated from bone marrow of WT mice were incubated for 4 hours with HSV-2 infected Vero cells (targets) that had been pretreated with control or ΔgD-2 immune serum. (A) Representative flow plots of intracellular granzyme B and perforin (after addition of protein transport inhibitor cocktail at 2 hours) in indicated immune cell populations isolated from bone marrow. (B) Representative flow plots of CD107a expression and intracellular granzyme B of indicated immune cell populations incubated with HSV-infected Vero cells treated with ΔgD-2 serum or control serum.

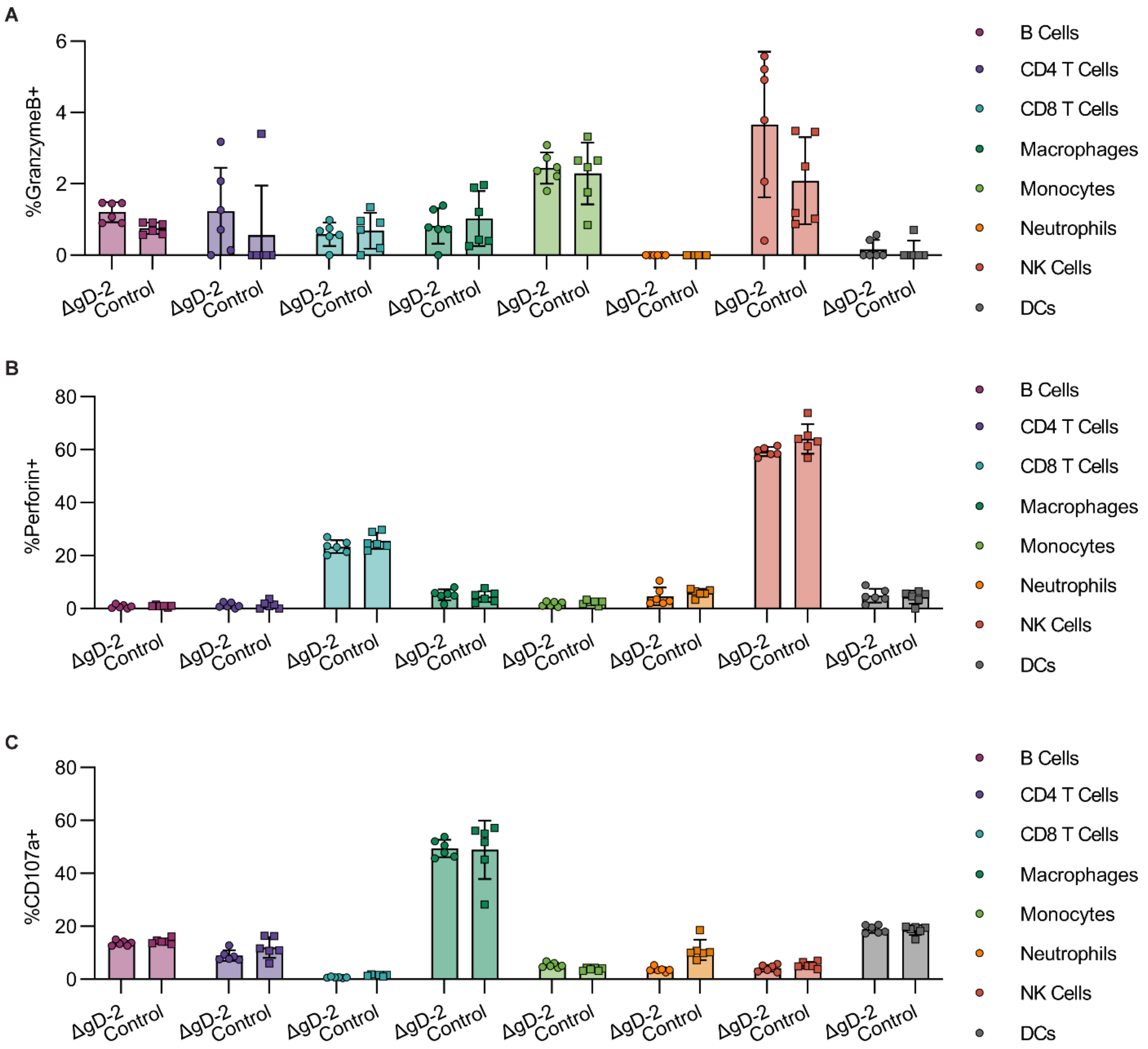

Figure S4. Total granzyme, perforin, and CD107a expression following coculture experiments. WT bone marrow was dissociated into single cell suspensions and cocultured with HSV-2 infected Vero cells incubated with either control or ΔgD-2 immune serum. Two hours after incubation cells were treated with a protein transport inhibitor cocktail (eBioscience) and incubated another two hours before staining to assess production of (A) granzyme B or (B) perforin. Alternatively, an antibody for CD107a was added prior to coculturing the immune cells with the targets and analyzed for CD107a cell surface expression after a 4-hour incubation.

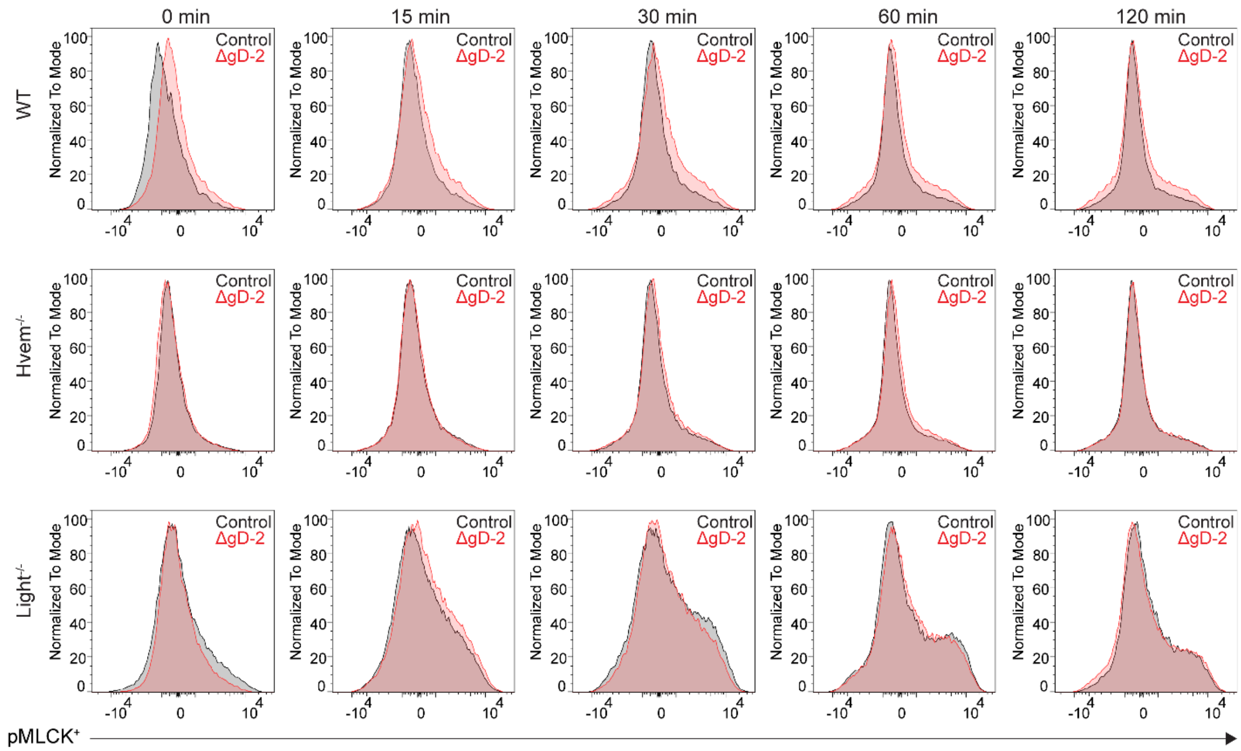

Figure S5. Representative flow plots for pMLCK expression. WT, *Hvem*$^{-/-}$, or *Light*$^{-/-}$ neutrophils were cocultured with HSV-infected Vero cells in the presence of ΔgD-2 (red) or control (black) serum for 0, 15, 30, 60, or 120 minutes. Following incubation, cells were fixed, permeabilized, and stained for phosphorylation of MLCK.

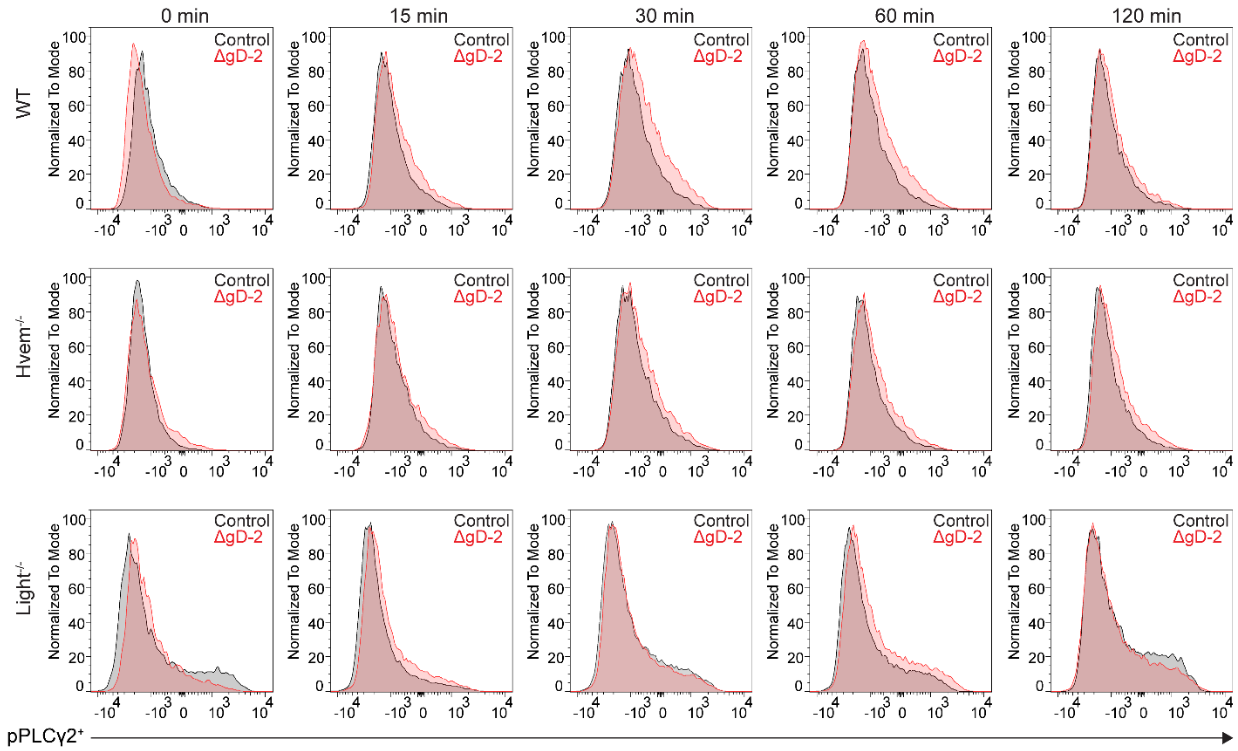

Figure S6. Representative flow plots for pPLCγ2 expression. WT, *Hvem*$^{-/-}$, or *Light*$^{-/-}$ neutrophils were cocultured with HSV-infected Vero cells in the presence of ΔgD-2 (red) or control (black) serum for 0, 15, 30, 60, or 120 minutes. Following incubation, cells were fixed, permeabilized, and stained for phosphorylation of PLCγ2.

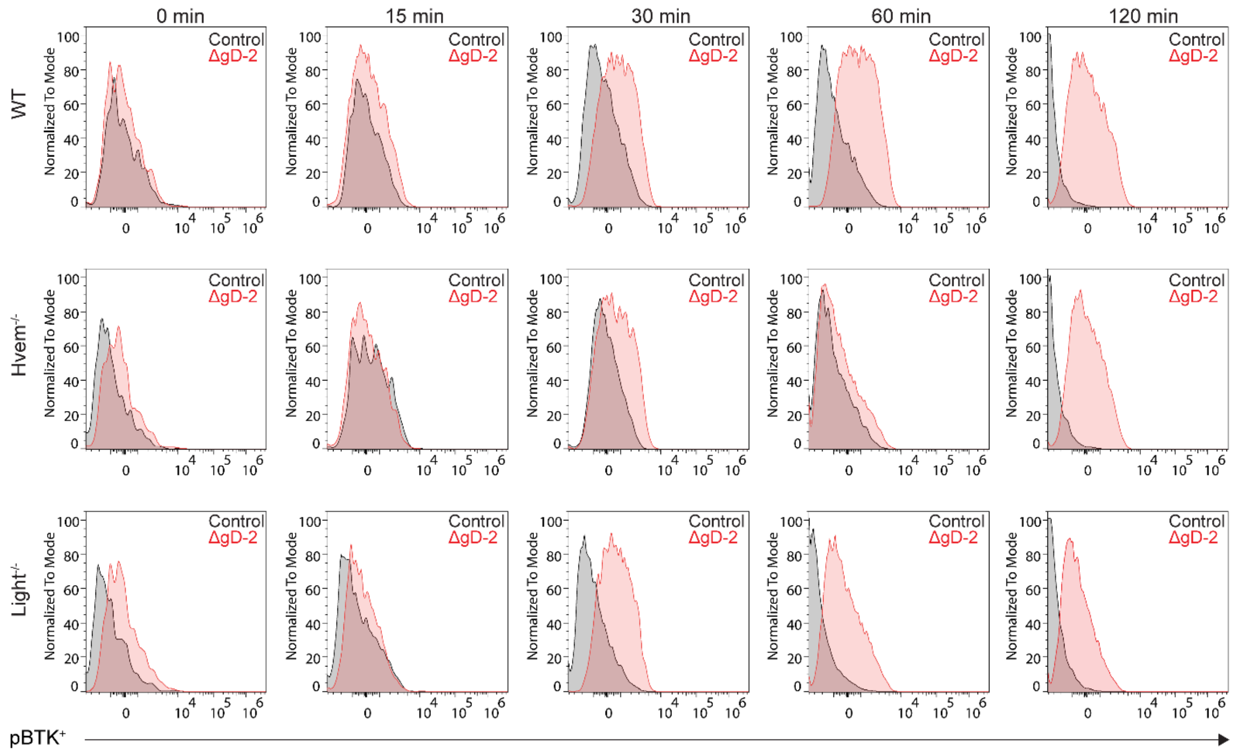

Figure S7. Representative flow plots for pBTK expression. WT, *Hvem*$^{-/-}$, or *Light*$^{-/-}$ neutrophils were cocultured with HSV-infected Vero cells in the presence of ΔgD-2 (red) or control (black) serum for 0, 15, 30, 60, or 120 minutes. Following incubation, cells were fixed, permeabilized, and stained for phosphorylation of BTK.

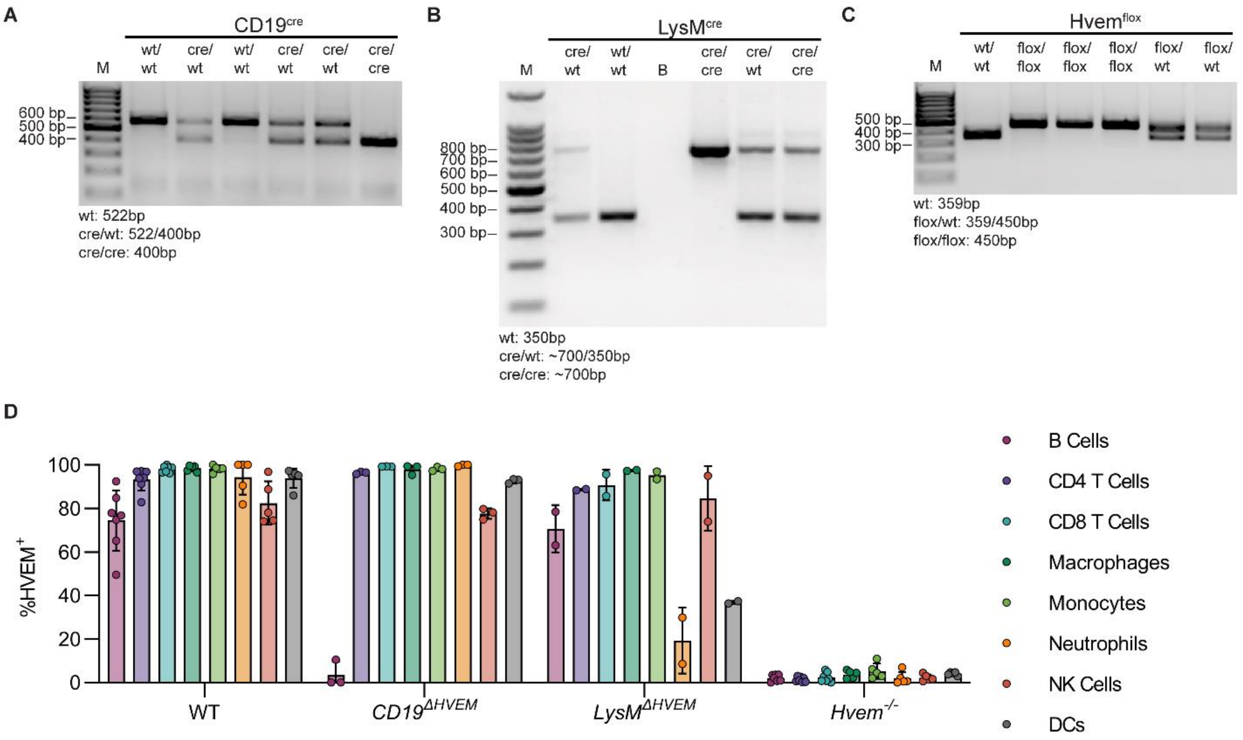

Figure S8. Confirming cell-specific HVEM deletion in *CD19$^{\Delta HVEM}$* and *LysM$^{\Delta HVEM}$* strains. Tail biopsies were collected from 3-week-old *CD19$^{\Delta HVEM}$* and *LysM$^{\Delta HVEM}$* mice and digested for DNA. DNA sequences for the Cre or LoxP insertions were amplified by PCR and analyzed by agarose gel electrophoresis. Representative data is shown for (A) *CD19^{Cre}*, (B) *LysM^{Cre}*, and (C) *Hvem^{flox}* with indicated base pair length for WT and transgene insertions. (D) Spleens were isolated from WT, *CD19$^{\Delta HVEM}$*, *LysM$^{\Delta HVEM}$*, and *Hvem$^{-/-}$* mice and dissociated into single cell suspensions. Cells were stained for immune cell subpopulations and HVEM expression.

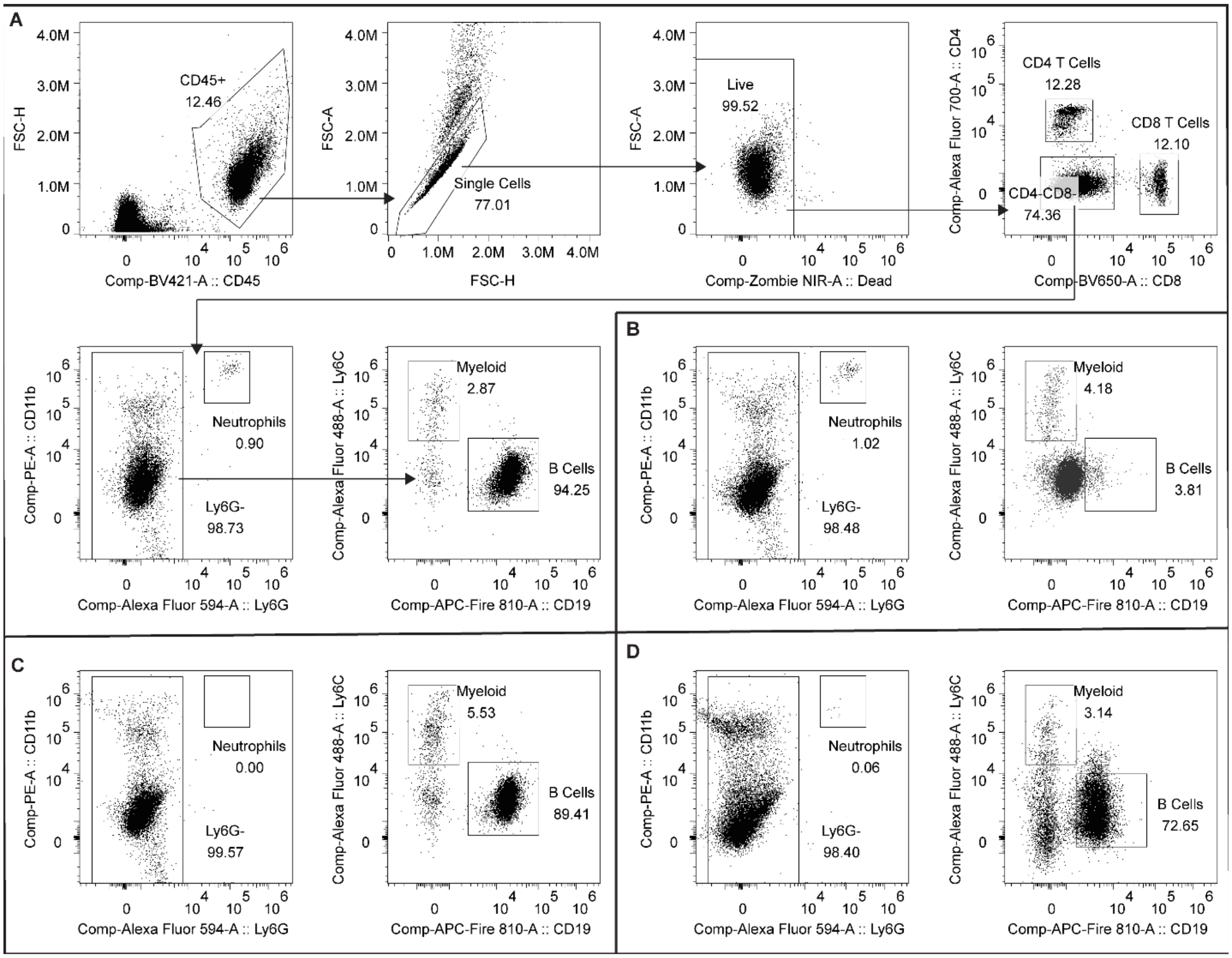

Figure S9. Depletion of specific immune cell populations following antibody injection. Blood was collected one-day post-intraperitoneal injection of depleting antibodies or isotype control. Following RBC lysis, immune cells were stained for flow cytometry to assess depletion efficacy. Representative flow plots are shown for (A) isotype control, (B), anti-CD19, (C) anti-Ly6G, and (D) anti-GR1.

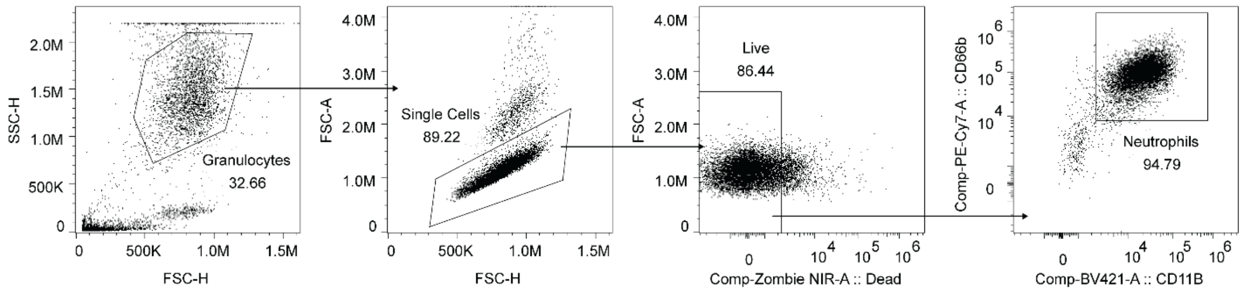

Figure S10. Gating strategy for human neutrophils. Human granulocytes were isolated following removal of PBMCs by Percoll gradient and red blood cell lysis. Granulocytes were identified by $FSC^{hi}SSC^{hi}$, gated on live single cells, and confirmed by expression of CD11b and CD66b.

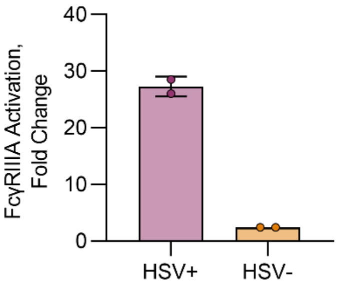

Figure S11. FcRIIIA activation of human donor serums. Blood was collected from human donors with known HSV serostatus for serum isolation. Serum was tested for activation of FcγRIIIA with Promega's ADCC Reporter Bioassay.

Video S1. Trogocytosis of target cell membrane by WT neutrophils. Live cell imaging of WT mouse neutrophils incubated with DiI- and Calcein-am-labeled HSV-infected Vero cells opsonized with ΔgD-2 immune serum (1:5, E:T ratio). Recording started 15 minutes after initiation of the coculture and performed at 30 s/frame and played back at 10 frames/s.

Video S2. Impaired trogocytosis of target cell membrane by *Hvem*$^{-/-}$ neutrophils. Live cell imaging of *Hvem*$^{-/-}$ mouse neutrophils incubated with DiI- and Calcein-am-labeled HSV-infected Vero cells opsonized with ΔgD-2 immune serum (1:5, E:T ratio). Recording started 15 minutes after initiation of the coculture and performed at 30 s/frame and played back at 10 frames/s.

Video S3. No trogocytosis of target cell membrane by *FcγRIV*$^{-/-}$ neutrophils. Live cell imaging of *FcγRIV*$^{-/-}$ mouse neutrophils incubated with DiI- and Calcein-am-labeled HSV-infected Vero cells opsonized with ΔgD-2 immune serum (1:5, E:T ratio). Recording started 15 minutes after initiation of the coculture and performed at 30 s/frame and played back at 10 frames/s.
