# Supplementary figures and images for: HVEM-LIGHT signaling promotes antibody-dependent neutrophil FcγR-mediated trogocytosis against herpes simplex virus infection
Source: J Clin Invest. 2026 Jun 4;136(14):e203771. doi: 10.1172/JCI203771 (PMC13367962; doi:10.1172/JCI203771)

# Full unedited gel for Figure S2

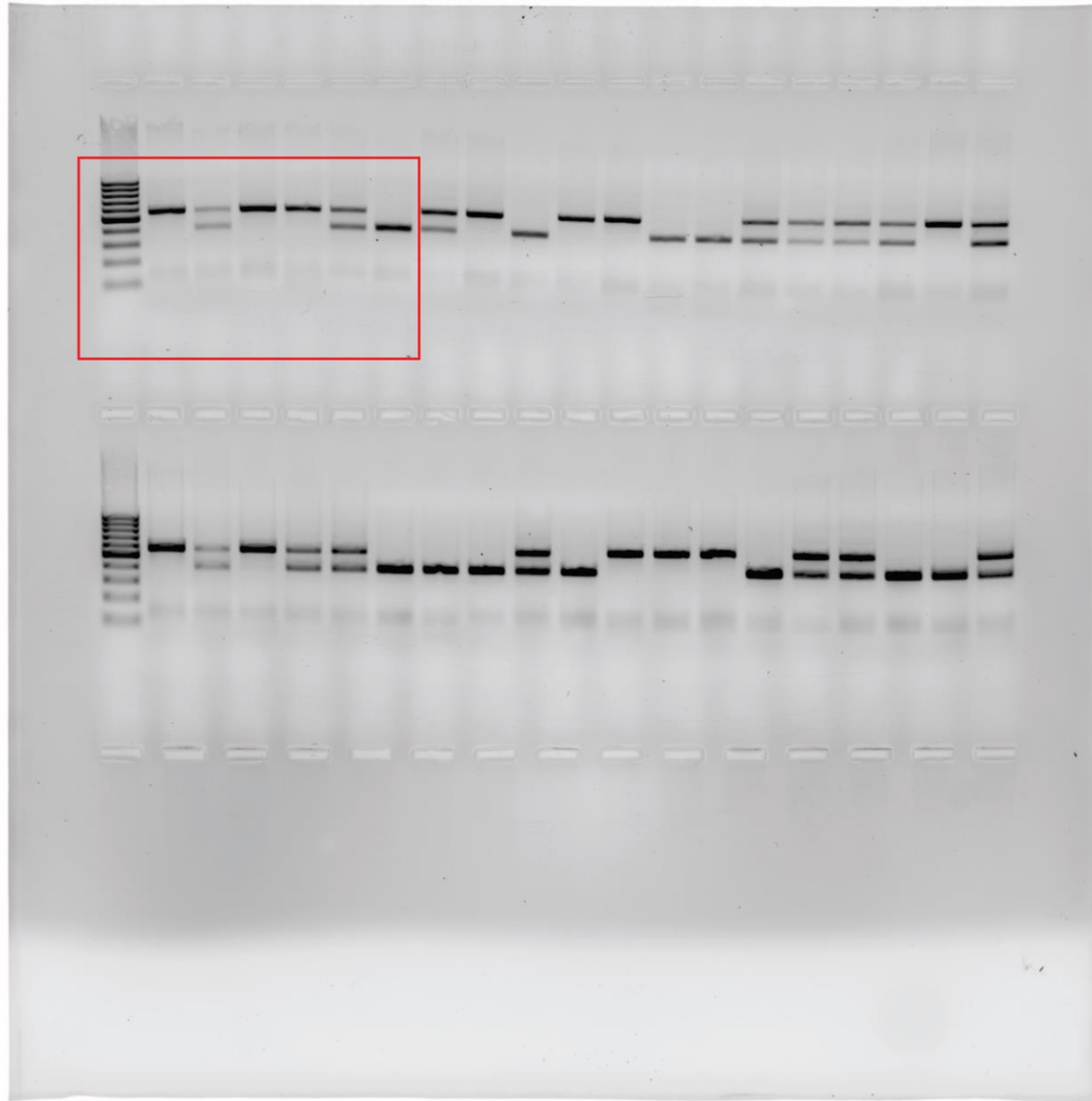

# Full unedited gel for Figure S2

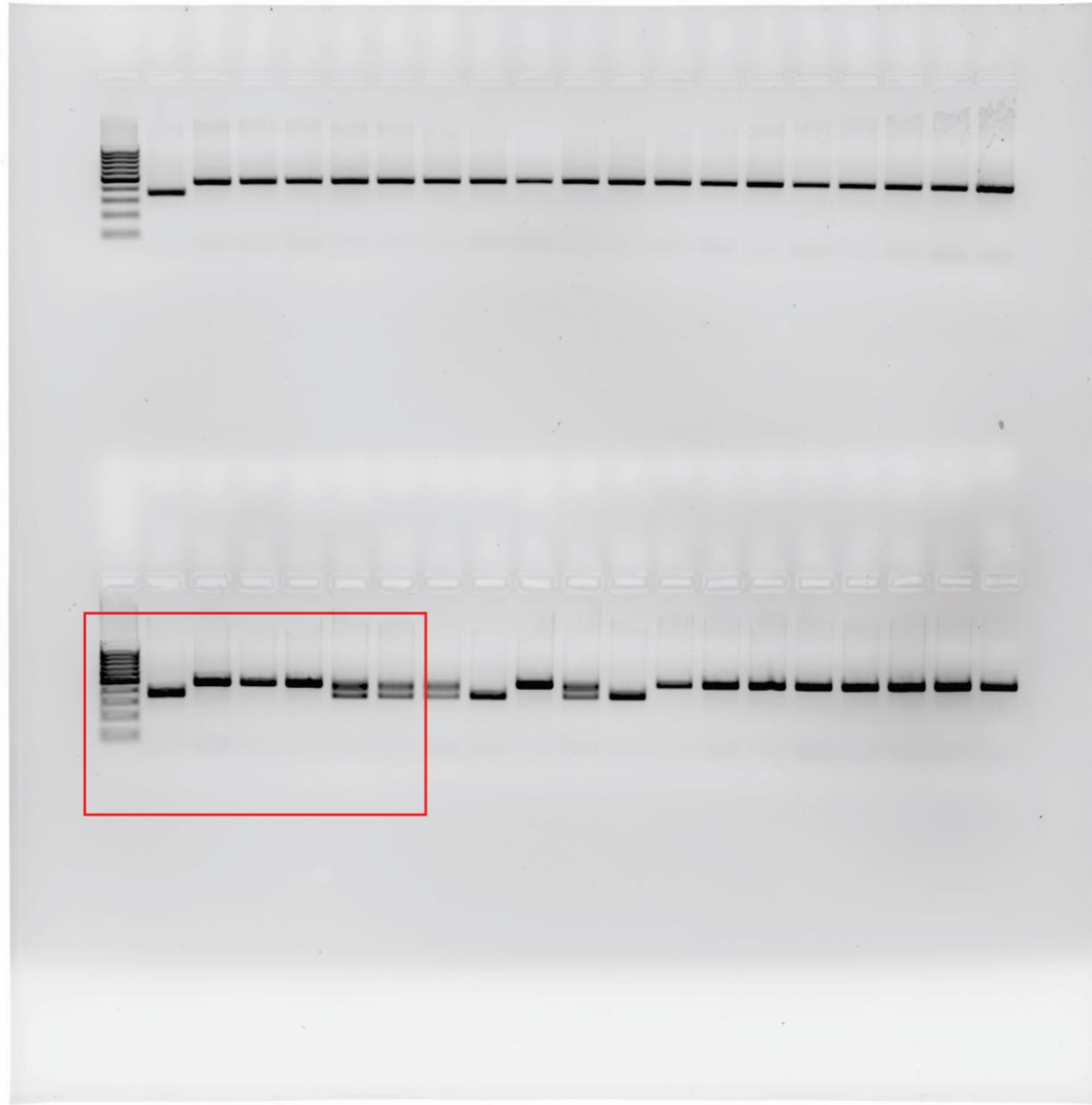

# Full unedited gel for Figure S2

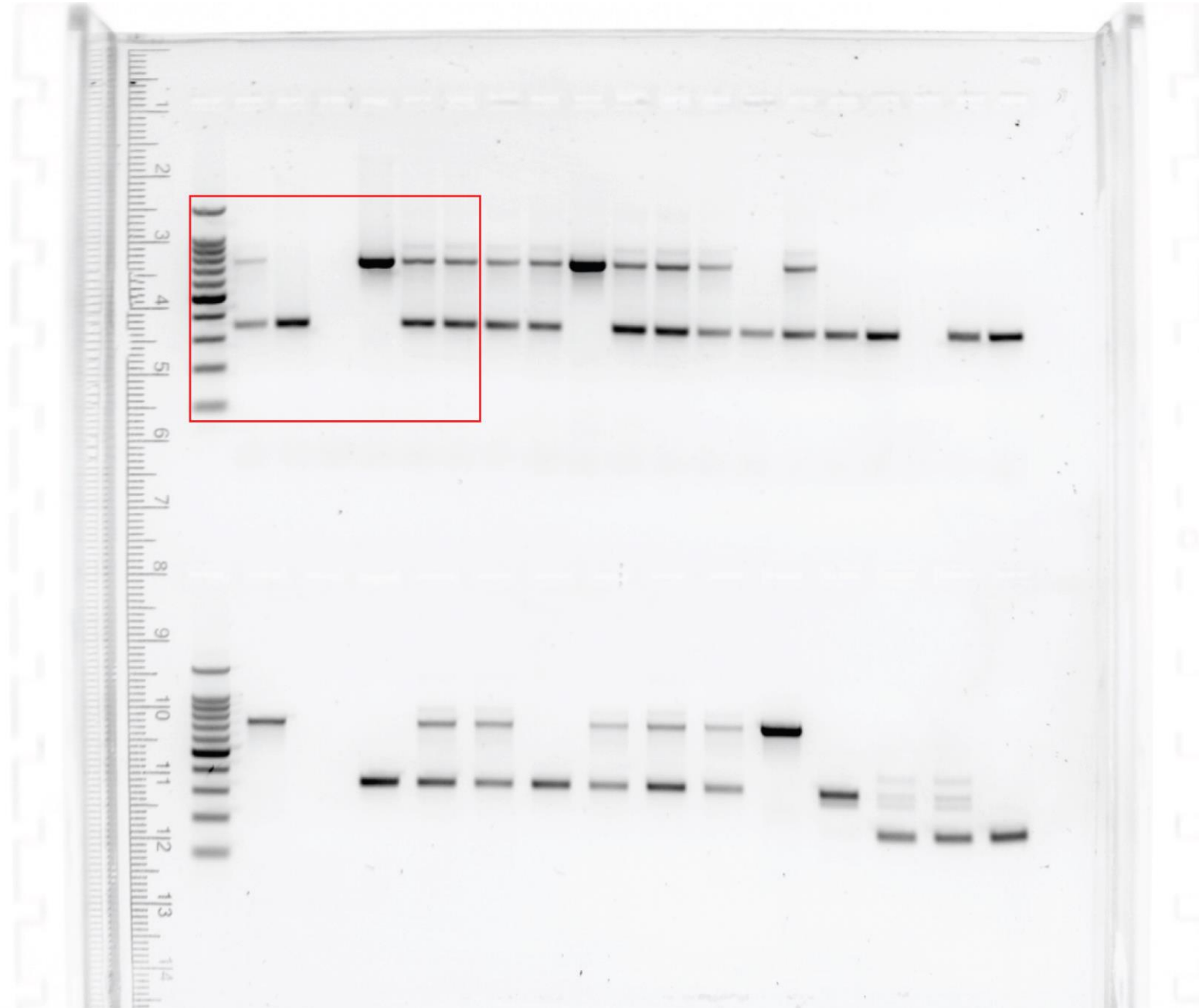

Supplement: Unedited blot and gel images [file jci-136-203771-s317.pdf]
